# Quantitative Trait Locus Mapping for Flowering Time in a Lowland × Upland Switchgrass Pseudo‐F_2_ Population

**DOI:** 10.3835/plantgenome2017.10.0093

**Published:** 2018-07-01

**Authors:** Carl‐Erik Tornqvist, Megan Taylor, Yiwei Jiang, Joseph Evans, C. Robin Buell, Shawn M. Kaeppler, Michael D. Casler

**Affiliations:** ^1^ Dep. of Agronomy and DOE Great Lakes Bioenergy Research Center Univ. of Wisconsin Madison WI 53706; ^2^ DNASTAR Madison WI 53705; ^3^ Dep. of Agronomy and DOE Great Lakes Bioenergy Research Center Univ. of Wisconsin Madison WI 53706; ^4^ Dep. of Plant Biology and DOE Great Lakes Bioenergy Research Center Michigan State Univ. East Lansing MI 48824; ^5^ DuPont Pioneer Johnston IA 50131; ^6^ Dep. of Agronomy Purdue Univ. West Lafayette IN 47907; ^7^ Dep. of Plant Biology and DOE Great Lakes Bioenergy Research Center Michigan State Univ. East Lansing MI 48824; ^8^ USDA‐ARS, U.S. Dairy Forage Research Center Madison WI 53706

## Abstract

Flowering is an important developmental event in switchgrass (*Panicum virgatum*), as the time to complete the life cycle affects overall biomass accumulation. The objective of this study was to generate a linkage map using single nucleotide polymorphism (SNP) markers to identify quantitative trait loci (QTL) associated with flowering time. A pseudo‐F_2_ population was created by crossing two siblings derived from an initial cross between the lowland population Ellsworth and the upland cultivar Summer. Heading and anthesis dates were collected for 2 yr at two locations: DeKalb, IL and Lafayette, IN. Nine QTL for flowering time were detected, two of which were heading‐associated, four anthesis‐associated, and three associated with both heading and anthesis. One QTL on linkage group (LG) 2a was detected for heading and anthesis in each location and year when environments were analyzed separately, and in a combined analysis across both locations and years. The effect on heading and anthesis of the QTL on LG 2a ranged from 4 to 13 and 5 to 9 d, respectively, depending on environment. Our findings validate QTL for switchgrass flowering time from previous research and identified additional QTL. Based on the switchgrass reference genome version 1.1, flowering time gene homologs reside near the LG 2a QTL and include PSEUDO RESPONSE REGULATOR 5, SUPPRESSOR OF FRIGIDA 4, and APETALA 1, respectively involved in the circadian clock, vernalization, and floral meristem identity. Markers linked to the QTL can be used to improve the efficiency of breeding switchgrass for delayed flowering to increase biomass yield.

**Abbreviations**
AD: anthesis date
AED: augmented experimental design
DOY: day of year
EBLUE: experimental best linear unbiased estimate
GDD: growing degree days
HD: heading date
LG: linkage group
LOD: logarithm of odds
QTL: quantitative trait loci
SNP: single nucleotide polymorphism
TY&TL: two years and two locations

## Core Ideas

- Phenotypic data for two indicators of flowering time were assessed at two locations for two years.
- A genetic linkage map was created from a switchgrass pseudo‐F_2_ population.
- Associations between phenotypic data and the linkage map were analyzed.
- Nine quantitative trait loci for flowering time were identified in the pseudo‐F_2_ population.
- One significant quantitative trait locus was stably expressed in multiple environments.

Switchgrass (*Panicum virgatum*) is a C4 perennial prairie grass that is native to North America. Due to its drought resistance, low‐input requirements, perenniality, and high biomass yield, switchgrass is a priority crop for use as a feedstock for cellulosic biofuel, as deemed by the U.S. Department of Energy (Sanderson et al., 2006). Switchgrass displays tremendous diversity in morphology and growth habitat, due to genetics, and expression of allelic variation in response to environmental drivers such as photoperiod, temperature, and precipitation. There are two ecotypes of switchgrass: upland and lowland. Upland switchgrass is adapted to northern climates, and is relatively cold‐tolerant and early flowering. Lowland switchgrass, on the other hand, is found naturally in southern climates, generally not cold‐tolerant when grown in northern climates, and is late flowering (Casler, 2012).

Lowland switchgrass grown in southern environments can easily reach biomass yields of 20 Mg/ha, sufficient for sustainable production of biomass for conversion to energy (Casler, 2012, Taliaferro, 2002). In northern zones, however, the biomass yields of upland switchgrass fall short of the southern‐grown lowland varieties due to the early flowering time, which restricts the vegetative growth phase (Casler, 2012, Casler and Vogel, 2014). Therefore, efforts to improve the biomass yield of northern‐grown switchgrass are focused on elucidating the flowering time pathway to develop markers that can be used to breed for late‐flowering and cold‐tolerant varieties. To date, only a few studies have focused on elucidating the flowering pathway in switchgrass. These studies have identified QTL associated with reproductive maturity (Dong et al., 2015, Milano et al., 2016), entailed genome‐wide analyses (Grabowski et al., 2016), or analyzed expression of orthologs of known flowering time genes (Niu et al., 2016, Tornqvist et al., 2017). Previous studies of QTL used lowland populations derived by either self‐pollination or crosses between lowland genotypes, except for the Milano et al. (2016) study that used a 4‐way population derived from two initial crosses, each between an upland and lowland genotype. In this study, we developed a pseudo‐F_2_ population derived from a cross between an upland and lowland variety to identify loci associated with two measures of flowering: heading and anthesis. Using single nucleotide polymorphism (SNP) markers from exome‐capture sequencing, a genetic linkage map was constructed for QTL analysis. The pseudo‐F_2_ population was planted at two locations and phenotypic data was collected for 2 yr at each site. Results of QTL analysis should enable development of molecular markers for breeding of both upland and lowland switchgrass that has delayed flowering and, therefore, increased biomass yield (Casler, 2012, Casler et al., 2012). Identification of chromosomal regions that contain QTL regulating flowering time will allow the development of marker‐assisted breeding schemes, allowing pre‐screening of thousands of genotypes in the glasshouse before establishment of expensive breeding nurseries in the field (Hayes et al., 2013, Hayward et al., 1994, Resende et al., 2013, 2014). The goals of this study, therefore, were to: (1) generate a SNP‐based linkage map, (2) identify genomic regions in a switchgrass hybrid population associated with two flowering time‐related traits, heading date and anthesis date, and (3) compare the putative QTL associations against previous results.

## MATERIALS AND METHODS

### Plant Materials and Planting Design

The initial cross of B901, a plant from the population Ellsworth (originating from a prairie remnant near Ellsworth, KS), with S041, a plant from the cultivar Summer, was made in the glasshouse in 2012. Seeds were collected from female plant B901 and seedlings were germinated in February 2013 to generate F_1_ plants for a second crossing season in 2013. Two random F_1_ individuals (numbered BxS1 and BxS7) were used to generate the ELLSU‐17 pseudo‐F_2_ cross. Following the F_2_ seed harvest, seeds were first imbibed in ddH_2_O between two pieces of filter paper in a covered Petri dish at 4°C for 2 wk. After imbibition, seeds were surface sterilized with 5% Clorox bleach and rinsed three times with ddH_2_O. To initiate germination, seeds were arranged on sterilized filter paper saturated with 60mM hydrogen peroxide in a sealed Petri dish and placed in an incubator at 30 to 35°C, with continuous light for 4 to 7 d, until all or most of the seeds germinated. After germination, seedlings were transplanted to containers (2.5 cm diameter) containing fine soil mix and grown in a greenhouse under natural and supplemented light. After two tillers had developed, seedlings were split into two vegetative ramets and allowed to grow for at least 2 wk, until at least one new tiller formed on each ramet. Each of the two ramets was then split again, resulting in four ramets for each F_2_ individual. However, not all individuals grew fast enough for generation of four ramets each and, therefore, for some individuals there were only two or three ramets each. In addition, multiple vegetative ramets of the parents and grandparents were generated to use as checks in the field.

Vegetative ramets for each individual were divided into two groups for planting in two field locations: DeKalb, IL (41.77 N) and Lafayette, IN (40.43 N). The two sites were chosen to simplify travel arrangements, while being located far enough south of Madison to ensure adequate winter survivorship, which is unreliable for lowland genotypes in Wisconsin (Casler and Vogel, 2014). The spatial layout of each field followed an augmented experimental design (AED) with 10 blocks, similar to that shown in Casler et al. (Casler et al., 2015). A total of 342 F_2_ genotypes, with 1–2 replications each were randomly assigned across the 10 blocks at each location, with a group of eight randomly arranged check plants (parents, grandparents, and two siblings of B×S1 and B×S7) centered in each of the 10 blocks, for a total of 560 plants at DeKalb and 555 plants at Lafayette. In July of 2014, the populations were transplanted to each field and irrigated as needed for successful establishment. The spacing between adjacent plants was 0.9 m.

### Phenotypic Data Collection

All plants were fertilized with 100 kg N/ha in April 2015 and 2016. Weed control was facilitated with a pre‐emergence herbicide treatment in April of each year, as described by (Casler, 2005) and by hand weeding later in the year. Beginning with the appearance of the first panicle at each location, all plants were scored for heading or anthesis, in general, every other day. Scoring continued until the last plant reached anthesis in 2016, or until September 28 in 2015. Heading, in this study, is defined as the stage at which at least 50% of the tillers of a plant are estimated to have an emerging panicle. Likewise, anthesis is defined as the stage at which at least 50% of the panicles have at least one floret with visible anthers. Heading and anthesis dates were recorded as day of year (DOY). For both years, one person at each location conducted scoring of heading and anthesis.

### Genotypic Data

DNA was extracted from 20 mg of lyophilized leaf tissue from greenhouse‐grown seedlings, following the CTAB method (Doyle and Doyle, 1990). Genotyping by exome‐capture sequencing was performed at the University of Wisconsin Biotechnology Center as described in Evans et al. (Evans et al., 2015). Single nucleotide polymorphism (SNP) markers that had been subsequently identified in Evans et al. (2015) were used to create a SNP matrix for the ELLSU‐17 population. All SNP genotypes were called using a custom script in the R programming language (R Core Team, 2014). Using the same R script, SNPs were further filtered by genotype segregation patterns in the F_2_ generation. Since the original SNP matrix was based on polymorphic markers identified in a panel of over 500 diverse switchgrass genotypes, most (> 99%) of the SNPs were monomorphic in the ELLSU‐17 population. Based on chi square analysis, the majority of polymorphic SNPs displayed no segregation distortion (*p* > 0.05). Since there were many more SNPs with non‐distorted segregation patterns, only SNPs of this type were used for linkage mapping. A final filtering step removed SNPs within 5kb of each other, by keeping the marker that had the least missing data.

### Linkage Map Construction

A total of 1230 SNPs were entered as “CP” type population for linkage analysis in JoinMap version 4.1 (Van Ooijen, 2006), with segregation types <hk×hk>, or F_2_‐type, segregating 1:2:1, and <lm×ll> and <nn×np>, both BC1‐type, segregating one‐to‐one. A total of 1223 SNPs were subsequently grouped into linkage groups and ordered according to the user manual, with the following calculation options. For grouping, the independence logarithm of odds (LOD) parameter was used, with an LOD threshold ranging from 2.0 to 14.0 and a step of 1.0. For marker ordering, the regression mapping algorithm was used, with parameters as follows: use linkages with a recombination frequency smaller than 0.400 and a LOD larger than 1.00, jump threshold of 5.00, and ripple = 1. Kosambi's mapping function was selected for calculating genetic distances and a third round was employed. Map charts were generated in JoinMap (Kyazma, Netherlands).

### Statistical Data Analysis

Broad sense heritability estimates were based on the equation:

$$H^{2}=\frac{V_{g}}{V_{g}+\frac{V_{gl}}{1}+\frac{V_{gy}}{y}+\frac{V_{gly}}{y1}+\frac{V_{e}}{2\left(y1\right)}}$$

where variance components are: V_g_, genotype, V_gl_, genotype by location, V_gy_, genotype by year, V_gly_, genotype by year by location, and $V_{e}$, error. The terms “y” and “l” in the equation refer to the number of years and locations, respectively.

The SAS MIXED procedure (SAS Inst. Inc., Cary, NC) with model based on (Wolfinger et al., 1997) for AED, was used to calculate the experimental best linear unbiased estimates (EBLUEs) for each trait, heading date and anthesis date. The EBLUEs for heading and anthesis DOY were used for QTL analysis via the R/qtl package (Broman et al., 2003) in the R programming language (R Core Team, 2014). A 5 cM grid of pseudo‐markers, generated with the ‘calc.genoprob’ function, was used in QTL scans, with Haley‐Knott regression. The LOD penalties for main effects and interactions were calculated through 1000‐permutations of the ‘scantwo’ function. A multiple QTL model was implemented with the ‘stepwiseqtl’ function, with maximum 5 QTL, and forward/backward elimination, using the ‘refineqtl’ function. The 1.5 LOD drop interval and percent variance explained for each QTL were calculated using the ‘fitqtl’ function.

## RESULTS

### Phenotypic Variation

Genotypic variation and genotype × location and genotype × year interactions were significant for both heading and anthesis. Location variation was significant only for heading date, while year variation and location × year interaction were significant for anthesis only (Table 1).

**Table 1:** Mixed models analysis of variance for fixed effects associated with heading and anthesis dates in a pseudo‐F_2_ population in 2 yr (2015 and 2016) at two locations, DeKalb, IL and Lafayette, IN.

Heading date				
	df	Type III SS	F‐value	Significance
Year (Y)	1	128	2.49	
Location (L)	1	345	6.70	*
Y x L	1	189	3.67	
Block/Replicate	5	213	0.83	
Genotype (G)	131	40038	5.94	USA
G x Y	129	16985	2.56	USA
G x L	130	20257	3.03	USA
G x L x Y	122	10414	1.66	USA

Anthesis date				
	df	Type III SS	F‐value	Significance
Year (Y)	1	392	9.57	**
Location (L)	1	152	3.70	
Y x L	1	176	4.30	*
Block/Replicate	5	358	1.75	
Genotype (G)	125	28977	5.66	USA
G x Y	124	14292	2.81	USA
G x L	124	17522	3.45	USA
G x L x Y	117	10359	2.16	USA

* Significant at the 0.05 probability level.

** Significant at the 0.01 probability level.e

USA Significant at the 0.001 probability level.

Estimated broad sense heritability was medium to high, at 0.62 and 0.60 for heading and anthesis, respectively.

The distribution of both heading and anthesis dates was normal and continuous for all environments. The range of F_2_ values extended beyond that of the parent mean values, indicating transgressive segregation. Mean heading and mean anthesis dates at DeKalb in 2015 were later than in Lafayette, by over 20 d. However, in 2016 mean heading and anthesis dates, although still later for DeKalb, differed by less than 10 d. Parents at DeKalb were also consistently later in heading and anthesis than at Lafayette, but by a smaller margin than for the F_2_ population. At both locations, for both years, parent B×S1 reached heading and anthesis before parent B×S7 (Table 2).

**Table 2:** Mean, minimum, maximum, and standard deviation (SD) of heading and anthesis dates in a pseudo‐F_2_ population, along with mean values for its two parents in 2 yr, 2015 and 2016, at two locations (TY&TL), DeKalb and Lafayette and in a combined analysis, TY&TL.

Location	Statistic	Heading date†		Anthesis date†	
		2015	2016	2015	2016
DeKalb	B×S1 mean	192	188	228	212
	B×S7 mean	204	193	239	217
	F_2_ mean	214	195	251	225
	F_2_ min.	183	182	203	204
	F_2_ max.	271	217	271	245
	F_2_ SD	17	6	14	9
Lafayette	B×S1 mean	182	180	206	213
	B×S7 mean	186	190	218	220
	F_2_ mean	186	189	218	220
	F_2_ min.	163	160	193	191
	F_2_ max.	218	212	246	238
	F_2_ SD	6	8	12	7
TY&TL	B×S1 mean	185		215	
	B×S7 mean	193		223	
	F_2_ mean	195		226	
	F_2_ min.	160		191	
	F_2_ max.	271		271	
	F_2_ SD	14		16	

† Day of year.

Across 2 yr and two locations, parent mean heading and anthesis dates both differed by 8 d, similar to 2016 means at both locations. Population mean heading and anthesis dates for the combined environments were also similar to 2016 means at each separate location (Table 2).

### Linkage Mapping and QTL Detection

A total of 1223 out of 1230 SNPs were grouped into 18 linkage groups, matching the 18 chromosomes of tetraploid switchgrass (Supplemental Fig. 1). The LGs were named according to the names of the chromosomes to which the SNPs were mapped on the switchgrass reference genome v1.1, with genome version 3 and later notation provided in this report in parentheses. Likewise, the SNPs were named according to the chromosome and position, in bases, based on the version 1.1 genome assembly. For example, the marker “*c1a_70873958” maps to base position 70,873,958 on chromosome 1a. The number of SNPs per LG ranged from 27 (LG 1b) to 96 (LG 2b). The total map length was 2453 cM, similar in size to three previously reported switchgrass linkage maps (Liu et al., 2012, Lowry et al., 2015, Milano et al., 2016), but larger than two published separate parental maps (Okada et al., 2010, Serba et al., 2013).

**Fig. 1:**
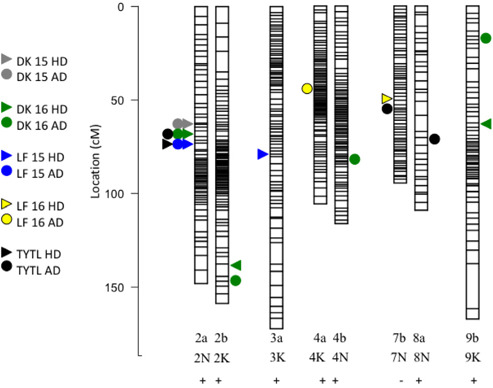
Flowering time QTL on selected linkage groups from a genetic linkage map of switchgrass. DK 15, DeKalb 2015; DK 16, DeKalb 2016; LF 15, Lafayette 2015; LF 16, Lafayette 2016; TYTL, Two Years & Two Locations; HD, Heading Date; AD, Anthesis Date. Linkage groups are labeled with genome v1 (top) and v3 (bottom) notation, along the horizontal axis. Black horizontal lines are locations of SNP markers. “+” and “‐” indicate effect direction based on expected contribution from the lowland allele.

Stepwise multiple QTL analysis of the two locations and years separately and combined revealed nine QTL affecting flowering time, present on LGs 2a (2N), 2b (2K), 3a (3K), 4a (4K), 4b (4N), 7b (7N), 8a (8N), and 9b (9K) (Fig. 1). Five QTL were detected for heading, while seven were detected for anthesis, with three QTL detected for both measures of flowering time. Each QTL explained approximately 8 to 24% of the phenotypic variation, with an average of 14% (Table 3, 4). For the 2 yr and two locations combined, the analysis detected one QTL for both heading and anthesis and two QTL for anthesis‐only. The three QTL reside on three LGs, 2a (2N), 7b (7N), and 8a (8N), respectively explaining approximately 8 and 11% (heading and anthesis), 9 and 10% of the phenotypic variation. Only one QTL, on LG 2a (2N), was identified in multiple environments, DeKalb in 2015, Lafayette in 2015, DeKalb in 2016, and using the combined EBLUEs across all environments. For all three environments and the combined two years and two locations (TY&TL), the QTL on LG 2a (2N) was associated with both heading and anthesis dates. Unexpectedly, none of the putative QTL identified for Lafayette in 2015 were identified in Lafayette in 2016, however the effect sizes and direction for the LG 2a (2N) locus in the Lafayette 2016 environment were on par with the environments with significant QTL. The QTL with the largest phenotypic variance explained (PVE), 24%, was located on LG 2b (2K), for heading date at DeKalb in 2016. The LG 2b (2K) QTL is also significant for anthesis date in the same environment, with a PVE of 14%. The QTL detected on LG 7b (7N), for both heading and anthesis, had an effect in the opposite direction than was expected, based on the contribution of the lowland allele. The LG 7b (7N) QTL was significant for heading at Lafayette in 2016 only and for anthesis in the 2 yr and locations combined. However, effect sizes and directions associated with this locus in a few other individual environments were similar to those observed at Lafayette in 2016, suggesting that they were not significant due to low statistical power (Table 3, 4).

**Table 3:** Quantitative trait loci detected and effect size and direction for heading in separate environments (DeKalb 2015 and 2016, Lafayette 2015 and 2016) and a combined environment over 2 yr and two locations (TYTL).

Heading	DeKalb 2015	DeKalb 2016	Lafayette 2015	Lafayette 2016	TYTL
LG† 2a (2N)					
Position‡	65.13	70.25	70.25	65.00	72.30
LOD§	4.42	5.79	5.38	ns¶	4.45
PVE#	12	16	12		8
Effect Size††	13	6	4	5	7
Effect Direction‡‡	+	+	+	+	+
LG 2b (2K)					
Position	140.00	140.00	140.00	140.00	140.00
LOD	ns	9.22	ns	ns	ns
PVE		24			
Effect Size	4	6	1	3	3
Effect Direction	+	+	+	+	+
LG 3a (3K)					
Position	81.06	81.06	81.06	81.06	81.06
LOD	ns	ns	4.94	ns	ns
PVE			11		
Effect Size	4	4	5	3	0
Effect Direction	+	+	+	+	n/a
LG 7b (7N)					
Position	48.80	48.80	48.80	48.80	48.80
LOD	ns	ns	ns	6.32	ns
PVE				14	
Effect Size	4	3	2	6	6
Effect Direction	–	–	–	–	–
LG 9b (9K)					
Position	63.25	63.25	63.25	63.25	63.25
LOD	ns	6.74	ns	ns	ns
PVE		18			
Effect Size	7	6	1	2	4
Effect Direction	+	+	+	+	+

† LG, linkage group.

‡ Centimorgans.

§ LOD, logarithm of odds.

¶ ns, not significant.

# PVE, phenotypic variance explained.

†† Mean difference, in days.

‡‡ Relative to the expected contribution of the lowland allele.

**Table 4:** Quantitative trait loci detected and effect size and direction for anthesis in separate environments (DeKalb 2015 and 2016, Lafayette 2015 and 2016) and a combined environment over 2 yr and two locations (TYTL).

Anthesis	DeKalb 2015	DeKalb 2016	Lafayette 2015	Lafayette 2016	TYTL
LG† 2a (2N)					
Position‡	65.00	70.00	70.00	70.00	70.00
LOD§	4.81	7.16	5.15	ns¶	6.08
PVE#	13	19	11		11
Effect Size††	9	9	9	5	9
Effect Direction‡‡	+	+	+	+	+
LG 2b (2K)					
Position	145.00	145.00	145.00	145.00	145.00
LOD	ns	5.34	ns	ns	ns
PVE		14			
Effect Size	3	4	2	1	4
Effect Direction	+	+	+	+	+
LG 4a (4K)					
Position	43.30	43.30	43.30	43.30	43.30
LOD	ns	ns	ns	6.43	ns
PVE				15	
Effect Size	7	4	4	5	4
Effect Direction	+	+	+	+	+
LG 4b (4N)					
Position	81.00	81.00	81.00	81.00	81.00
LOD	ns	5.29	ns	ns	ns
PVE		14			
Effect Size	6	7	1	3	5
Effect Direction	+	+	+	+	+
LG 7b (7N)					
Position	53.40	53.40	53.40	53.40	53.40
LOD	ns	ns	ns	ns	4.83
PVE					9
Effect Size	6	2	3	3	6
Effect Direction	–	–	–	–	–
LG 8a (8N)					
Position	71.10	71.10	71.10	71.10	71.10
LOD	ns	ns	ns	ns	5.07
PVE					10
Effect Size	0	4	6	1	5
Effect Direction	n/a	+	+	+	+
LG 9b (9K)					
Position	15.00	15.00	15.00	15.00	15.00
LOD	ns	5.07	ns	ns	ns
PVE		14			
Effect Size	7	5	0	1	2
Effect Direction	+	+	n/a	+	+

† LG, linkage group.

‡ Centimorgans.

§ LOD, logarithm of odds.

¶ ns, not significant.

# PVE, phenotypic variance explained.

†† Mean difference, in days.

‡‡ Relative to the expected contribution of the lowland allele.

## DISCUSSION

Flowering time is an important trait associated with biomass yield in switchgrass and other crops, such as sorghum, maize, and rice (Casler, 2014, Mullet et al., 2014, Russell and Stuber, 1983, Salam and Mackill, 1993). For switchgrass, utilization of germplasm with delayed flowering time has been partly responsible for increasing biomass yields by 35 to 50% during the past 20 yr of breeding in cold‐temperate regions of North America (Casler, 2012, 2014; Casler and Vogel, 2014). The past 20 yr of work has involved breeding within upland or lowland populations, but relatively little breeding effort within hybrids of the two ecotypes (Casler and Vogel, 2014). It is useful, however, to study mechanisms of flowering time in upland versus lowland germplasm to potentially parse out genetic pathways that act independently of the cold‐tolerance mechanisms. Ultimately, the goal is to retain the desirable cold hardiness of the upland ecotype while manipulating flowering in hybrid populations to retain the late flowering trait of the lowland ecotype. This was partially accomplished with the cultivar Liberty, but it required 18 yr from the time of the initial crosses until release (Vogel et al., 2014).

**Fig. 2:**
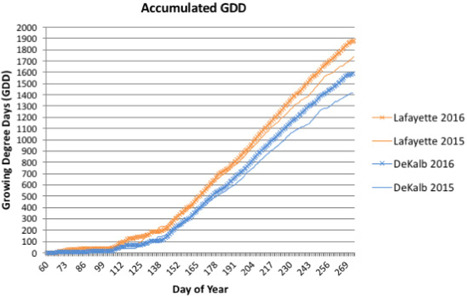
Accumulated growing degree days (GDD) at DeKalb and Lafayette in 2015 and 2016. GDD calculation based on equation from Mitchell et al. (1997).

Results of QTL analyses revealed several genomic regions controlling flowering time. Through linkage analysis, SNPs were grouped into 18 LGs, reflecting the 18 chromosomes of tetraploid switchgrass to which the SNPs had been previously mapped in the reference genome v1.1 (Evans et al., 2015). Only one of nine identified QTL was stably detected in multiple environments, indicating potentially strong genotype × year and/or genotype × location effects. Indeed, the genotype × year and genotype × location interactions are significant for both heading and anthesis (Table 1). Although DeKalb and Lafayette are separated by only 1.5 degrees of latitude and are both in USDA hardiness zone 5b, Lafayette received more precipitation than DeKalb (Supplemental Fig. 3), which could have affected early season growth leading to delayed development and subsequent flowering time at DeKalb. For the period between July and August 2014 (establishment season), the average precipitation at Lafayette was double that at DeKalb. In addition, the average precipitation at Lafayette in July 2015, the height of the growing season, was more than twice the amount at DeKalb. For July and August 2016, however, the average precipitation was about equal between the two locations. These differences in precipitation between the locations for the establishment year and 2015 may be a partial driver of the strong genotype × location interactions observed in this study. Mean daily temperatures between the two locations and years were not drastically different and photoperiods were identical (Supplemental Fig. 2). However, accumulated growing degree days (GDD) did differ between DeKalb and Lafayette (Fig. 2), which could be a major contributing factor to the G X E effect. For example, in both 2015 and 2016 the accumulated GDD at the F_2_ mean heading date (HD) and anthesis date (AD) for Lafayette were more than 100 more than the accumulated GDD for DeKalb at the same DOY. In addition, the accumulated GDD at the F_2_ mean HD and AD for DeKalb were on par with the accumulated GDD at the F_2_ mean HD and AD for Lafayette, suggesting that accumulated GDD may influence time to flowering more than just number of days after green up alone.

Three of the LGs in this study with significant flowering time QTL have also been identified as containing flowering time QTL in previous studies based on independent germplasm sources. (Dong et al., 2015) reported significant QTL for reproductive maturity on LGs 2b (2K) and 3a (3K), among others. Milano et al. (Milano et al., 2016) mapped flowering time QTL to LGs 2K and 4K, respectively analogous to LGs 2b and 4a in version 1.1 of the reference genome. It is worth noting, however, that in the two previous studies mentioned, the measurement of flowering time was not the same as in this study. Namely, Dong et al. (2015) scored the populations at different periods based on panicle development and Milano et al. (2016) used the date of the first emerging panicle as the heading date. These differences in the definition of flowering time could lead to differences in detected QTL. As evidenced by some QTL identified for only HD or only AD, heading and anthesis could be under separate genetic control. Along these lines, HD is directly related to time of transition from vegetative to reproductive phase, while AD relies on pathways affecting the physiology and development of the panicle after the phase transition.

While there was no mention of genotype × location or genotype × year effects in the Milano et al. (2016) study, Dong et al. (2015) reported significant genotype and genotype × location effects. Flowering time is clearly influenced by environmental factors and, therefore, selecting markers from QTL that have similar effect sizes across multiple environments will be necessary to efficiently breed late‐flowering switchgrass. For example, the QTL on LG 2a (2N) is significant in all environments except for Lafayette in 2016. However, the effect size of the locus at Lafayette in 2016 was similar to other effects in environments in which the QTL was significant. Therefore, the QTL on LG 2a (2N) is a worthwhile target for further investigation and development into potential breeding markers. In addition, the QTL on LG 4a (4K) has moderate effect sizes and while the QTL is not significant at DeKalb in 2015, the effect size in that environment, 7.4 d, is larger than that of the significant environment, Lafayette in 2016 (Table 3).

Orthologs of known flowering time genes reside within the 1.5 LOD confidence intervals of the QTL identified in this study. Examples on chromosome 2a (2N) include: PSEUDO‐RESPONSE REGULATOR 5 (PRR5) (Nakamichi et al., 2005), involved in the circadian clock and photoperiod detection, SUPPRESSOR OF FRIGIDA 4 (Kim and Michaels, 2006), a gene from *Arabidopsis* in the autonomous pathway that transcriptionally activates the floral repressor, FLOWERING LOCUS C, and APETALA 1 (Gustafson‐Brown et al., 1994), a floral meristem identity gene. Flowering time orthologs on chromosome 3a (3K) include: FRIGIDA ESSENTIAL 1 (Schmitz et al., 2005) and VERNALIZATION 5 (Greb et al., 2007), both in the vernalization pathway. On chromosome 4a (4K), orthologs of several high profile flowering time genes reside, including: FLOWERING LOCUS T (FT) (Kardailsky et al., 1999), TWIN SISTER OF FT (Yamaguchi et al., 2005), and CONSTANS (Putterill et al., 1995). Chromosome 7b (7N) harbors orthologs of LEAFY (Weigel et al., 1992), a floral meristem identity gene, and TERMINAL FLOWER 1 (Kobayashi et al., 1999), shown to act as a floral antagonist.

Two of the four QTL detected in this study reside on the same LGs as QTL identified in a previous study of switchgrass reproductive maturity in a lowland population (Dong et al., 2015), although whether the QTL confidence intervals from both studies contain the same genes, remains to be determined. In addition, the Dong et al. (2015) study used populations derived from lowlands only, whereas this study employed a population derived from a lowland × upland cross, which could contribute to the identification of QTL that differentiate the two ecotypes. With this lowland × upland cross, the expectation was that a mix of QTL effect directions would be observed. However, all but one QTL had positive effects, i.e., the lowland allele resulted in delayed flowering.

Studies of flowering time in mapping populations of other grasses, such as setaria, closely related to switchgrass, and sorghum, have yielded similar results to those presented here, in terms of genotypic variance, number of QTL detected, and percent of the phenotypic variance explained by the QTL (Mauro‐Herrera et al., 2013, Zou et al., 2012). While the mapping populations analyzed in the setaria and sorghum reports were inbred lines, however, unlike the pseudo‐F_2_ population in this study, the flowering time genes associated with the detected QTL in each study share similarities. For example, *Ma1* from sorghum is a PSEUDO‐RESPONSE REGULATOR‐like gene (Murphy et al., 2011) and CONSTANS was identified here and in the Mauro‐Herrera et al. (2013) setaria study. Through a systems approach and comparison across species, therefore, aspects of conserved mechanisms of genetic regulation of flowering among grasses become apparent.

## CONCLUSIONS

A SNP‐based linkage map was generated from a pseudo‐F_2_ population derived from hybrid (lowland × upland) F_1_ switchgrass parents. The population was grown for 2 yr at two locations to identify QTL for two flowering time‐related traits, heading date and anthesis date. Of the nine QTL revealed in separate environments, one was stably detected in multiple environments and in the combined TY&TL. The effects of all but one of the QTL were in the positive direction, which is expected if the late flowering allelic effect is contributed by the lowland allele. Even in environments in which a putative QTL was not significant, the effect sizes were on par with those of the significant QTL. For QTL with positive effects, markers within the QTL and 1.5 LOD confidence interval could be selected for, while QTL with negative effects could be selected against, in a breeding program to delay flowering time and increase biomass yield in switchgrass. Further fine mapping and functional analyses with mutants and/or genetically manipulated individuals of closely related species will enable choosing the best candidate genes for marker‐assisted breeding in switchgrass.

### Supplemental Material

Supplemental Fig. 1: Genetic linkage map of 1223 single nucleotide polymorphism (SNP) markers in 18 linkage groups (LGs). Map distances in Kosambi map units (cM) are shown on the left and marker names on the right of each LG, labeled with genome v1 notation and, in parentheses, v3 notation. Numbers in brackets denote segments of the labeled LG. Total map length was 2453 cM.

Supplemental Fig. 2: Daylength and daily mean temperatures at DeKalb and Lafayette in 2015, top, and 2016, bottom. Daylengths at DeKalb and Lafayette were identical.

Supplemental Fig. 3: Daily precipitation at DeKalb and Lafayette in 2015, top, and 2016, bottom.

### Conflict of Interest Disclosure

The authors declare that there is no conflict of interest.

## Supporting information

Supplemental Information
Supplemental File 1

Supplemental Information
Supplemental File 2

Supplemental Information
Supplemental File 3
