# Supplementary material for: Quantitative Trait Locus Mapping for Flowering Time in a Lowland × Upland Switchgrass Pseudo‐F2 Population
Source: Plant Genome. 2018 Jul 1;11(2):170093. doi: 10.3835/plantgenome2017.10.0093 (PMC12810072; doi:10.3835/plantgenome2017.10.0093)
Supplement: Supplementary file 1 [file TPG2-11-170093-s001.pdf]

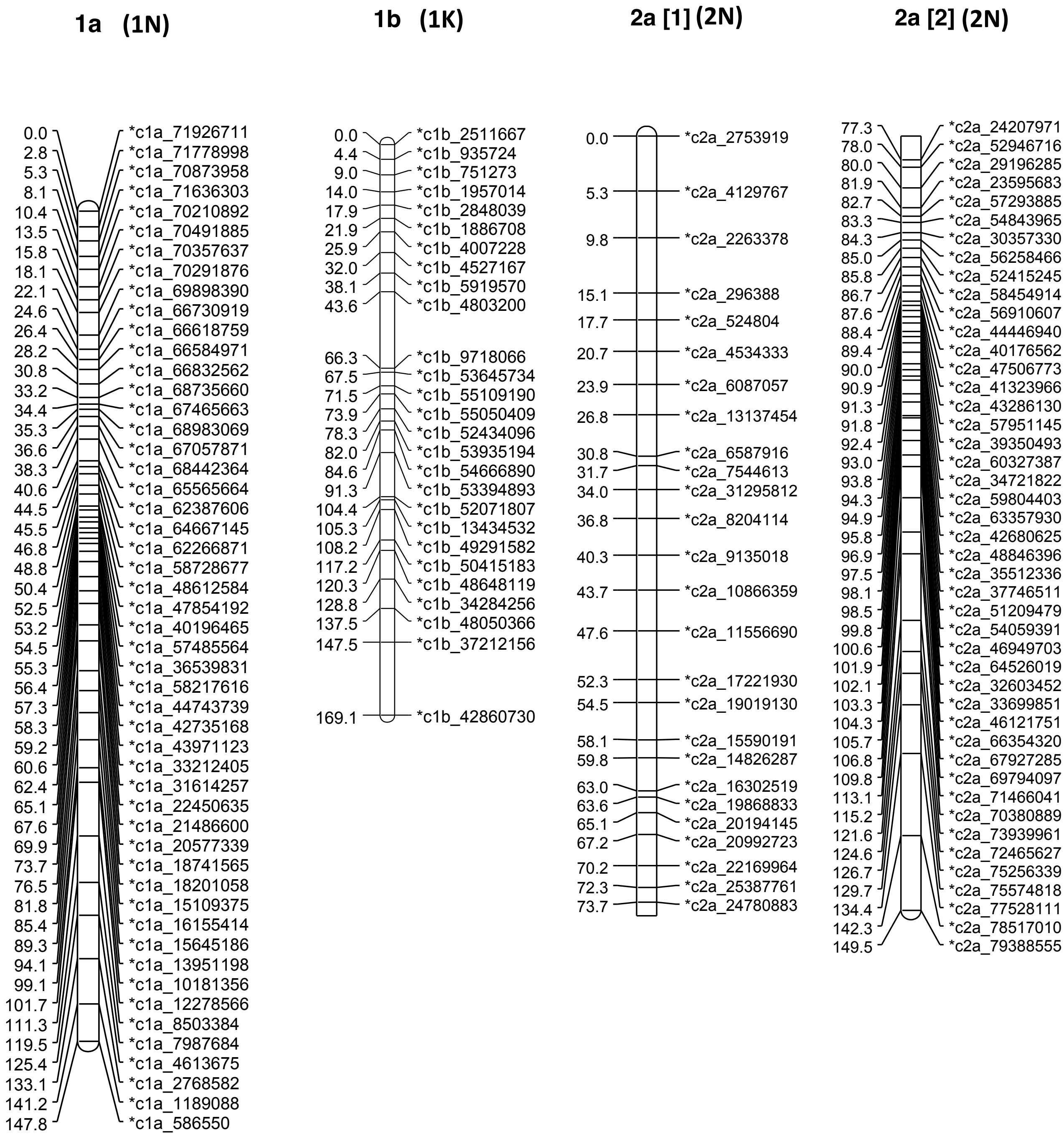

1a (1N)
0.0 *c1a_71926711
2.8 *c1a_71778998
5.3 *c1a_70873958
8.1 *c1a_71636303
10.4 *c1a_70210892
13.5 *c1a_70491885
15.8 *c1a_70357637
18.1 *c1a_70291876
22.1 *c1a_69898390
24.6 *c1a_66730919
26.4 *c1a_66618759
28.2 *c1a_66584971
30.8 *c1a_66832562
33.2 *c1a_68735660
34.4 *c1a_67465663
35.3 *c1a_68983069
36.6 *c1a_67057871
38.3 *c1a_68442364
40.6 *c1a_65565664
44.5 *c1a_62387606
45.5 *c1a_64667145
46.8 *c1a_62266871
48.8 *c1a_58728677
50.4 *c1a_48612584
52.5 *c1a_47854192
53.2 *c1a_40196465
54.5 *c1a_57485564
55.3 *c1a_36539831
56.4 *c1a_58217616
57.3 *c1a_44743739
58.3 *c1a_42735168
59.2 *c1a_43971123
60.6 *c1a_33212405
62.4 *c1a_31614257
65.1 *c1a_22450635
67.6 *c1a_21486600
69.9 *c1a_20577339
73.7 *c1a_18741565
76.5 *c1a_18201058
81.8 *c1a_15109375
85.4 *c1a_16155414
89.3 *c1a_15645186
94.1 *c1a_13951198
99.1 *c1a_10181356
101.7 *c1a_12278566
111.3 *c1a_8503384
119.5 *c1a_7987684
125.4 *c1a_4613675
133.1 *c1a_2768582
141.2 *c1a_1189088
147.8 *c1a_586550
1b (1K)
0.0 *c1b_2511667
4.4 *c1b_935724
9.0 *c1b_751273
14.0 *c1b_1957014
17.9 *c1b_2848039
21.9 *c1b_1886708
25.9 *c1b_4007228
32.0 *c1b_4527167
38.1 *c1b_5919570
43.6 *c1b_4803200
66.3 *c1b_9718066
67.5 *c1b_53645734
71.5 *c1b_55109190
73.9 *c1b_55050409
78.3 *c1b_52434096
82.0 *c1b_53935194
84.6 *c1b_54666890
91.3 *c1b_53394893
104.4 *c1b_52071807
105.3 *c1b_13434532
108.2 *c1b_49291582
117.2 *c1b_50415183
120.3 *c1b_48648119
128.8 *c1b_34284256
137.5 *c1b_48050366
147.5 *c1b_37212156
169.1 *c1b_42860730
2a [1] (2N)
0.0 *c2a_2753919
5.3 *c2a_4129767
9.8 *c2a_2263378
15.1 *c2a_296388
17.7 *c2a_524804
20.7 *c2a_4534333
23.9 *c2a_6087057
26.8 *c2a_13137454
30.8 *c2a_6587916
31.7 *c2a_7544613
34.0 *c2a_31295812
36.8 *c2a_8204114
40.3 *c2a_9135018
43.7 *c2a_10866359
47.6 *c2a_11556690
52.3 *c2a_17221930
54.5 *c2a_19019130
58.1 *c2a_15590191
59.8 *c2a_14826287
63.0 *c2a_16302519
63.6 *c2a_19868833
65.1 *c2a_20194145
67.2 *c2a_20992723
70.2 *c2a_22169964
72.3 *c2a_25387761
73.7 *c2a_24780883
2a [2] (2N)
77.3 *c2a_24207971
78.0 *c2a_52946716
80.0 *c2a_29196285
81.9 *c2a_23595683
82.7 *c2a_57293885
83.3 *c2a_54843965
84.3 *c2a_30357330
85.0 *c2a_56258466
85.8 *c2a_52415245
86.7 *c2a_58454914
87.6 *c2a_56910607
88.4 *c2a_44446940
89.4 *c2a_40176562
90.0 *c2a_47506773
90.9 *c2a_41323966
91.3 *c2a_43286130
91.8 *c2a_57951145
92.4 *c2a_39350493
93.0 *c2a_60327387
93.8 *c2a_34721822
94.3 *c2a_59804403
94.9 *c2a_63357930
95.8 *c2a_42680625
96.9 *c2a_48846396
97.5 *c2a_35512336
98.1 *c2a_37746511
98.5 *c2a_51209479
99.8 *c2a_54059391
100.6 *c2a_46949703
101.9 *c2a_64526019
102.1 *c2a_32603452
103.3 *c2a_33699851
104.3 *c2a_46121751
105.7 *c2a_66354320
106.8 *c2a_67927285
109.8 *c2a_69794097
113.1 *c2a_71466041
115.2 *c2a_70380889
121.6 *c2a_73939961
124.6 *c2a_72465627
126.7 *c2a_75256339
129.7 *c2a_75574818
134.4 *c2a_77528111
142.3 *c2a_78517010
149.5 *c2a_79388555

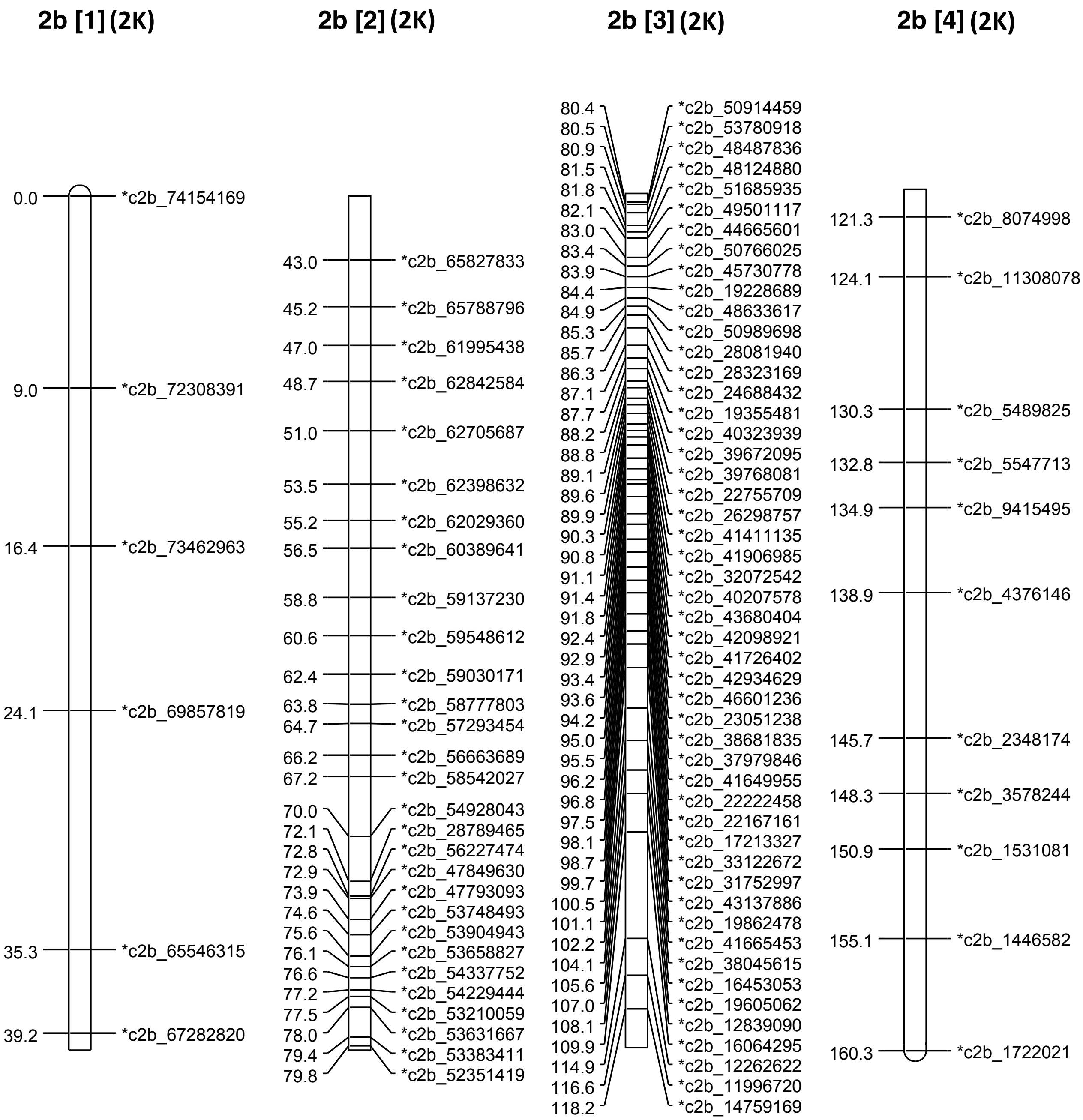
2b [1] (2K)
0.0 *c2b_74154169
9.0 *c2b_72308391
16.4 *c2b_73462963
24.1 *c2b_69857819
35.3 *c2b_65546315
39.2 *c2b_67282820
2b [2] (2K)
43.0 *c2b_65827833
45.2 *c2b_65788796
47.0 *c2b_61995438
48.7 *c2b_62842584
51.0 *c2b_62705687
53.5 *c2b_62398632
55.2 *c2b_62029360
56.5 *c2b_60389641
58.8 *c2b_59137230
60.6 *c2b_59548612
62.4 *c2b_59030171
63.8 *c2b_58777803
64.7 *c2b_57293454
66.2 *c2b_56663689
67.2 *c2b_58542027
70.0 *c2b_54928043
72.1 *c2b_28789465
72.8 *c2b_56227474
72.9 *c2b_47849630
73.9 *c2b_47793093
74.6 *c2b_53748493
75.6 *c2b_53904943
76.1 *c2b_53658827
76.6 *c2b_54337752
77.2 *c2b_54229444
77.5 *c2b_53210059
78.0 *c2b_53631667
79.4 *c2b_53383411
79.8 *c2b_52351419
2b [3] (2K)
80.4 *c2b_50914459
80.5 *c2b_53780918
80.9 *c2b_48487836
81.5 *c2b_48124880
81.8 *c2b_51685935
82.1 *c2b_49501117
83.0 *c2b_44665601
83.4 *c2b_50766025
83.9 *c2b_45730778
84.4 *c2b_19228689
84.9 *c2b_48633617
85.3 *c2b_50989698
85.7 *c2b_28081940
86.3 *c2b_28323169
87.1 *c2b_24688432
87.7 *c2b_19355481
88.2 *c2b_40323939
88.8 *c2b_39672095
89.1 *c2b_39768081
89.6 *c2b_22755709
89.9 *c2b_26298757
90.3 *c2b_41411135
90.8 *c2b_41906985
91.1 *c2b_32072542
91.4 *c2b_40207578
91.8 *c2b_43680404
92.4 *c2b_42098921
92.9 *c2b_41726402
93.4 *c2b_42934629
93.6 *c2b_46601236
94.2 *c2b_23051238
95.0 *c2b_38681835
95.5 *c2b_37979846
96.2 *c2b_41649955
96.8 *c2b_22222458
97.5 *c2b_22167161
98.1 *c2b_17213327
98.7 *c2b_33122672
99.7 *c2b_31752997
100.5 *c2b_43137886
101.1 *c2b_19862478
102.2 *c2b_41665453
104.1 *c2b_38045615
105.6 *c2b_16453053
107.0 *c2b_19605062
108.1 *c2b_12839090
109.9 *c2b_16064295
114.9 *c2b_12262622
116.6 *c2b_11996720
118.2 *c2b_14759169
2b [4] (2K)
121.3 *c2b_8074998
124.1 *c2b_11308078
130.3 *c2b_5489825
132.8 *c2b_5547713
134.9 *c2b_9415495
138.9 *c2b_4376146
145.7 *c2b_2348174
148.3 *c2b_3578244
150.9 *c2b_1531081
155.1 *c2b_1446582
160.3 *c2b_1722021

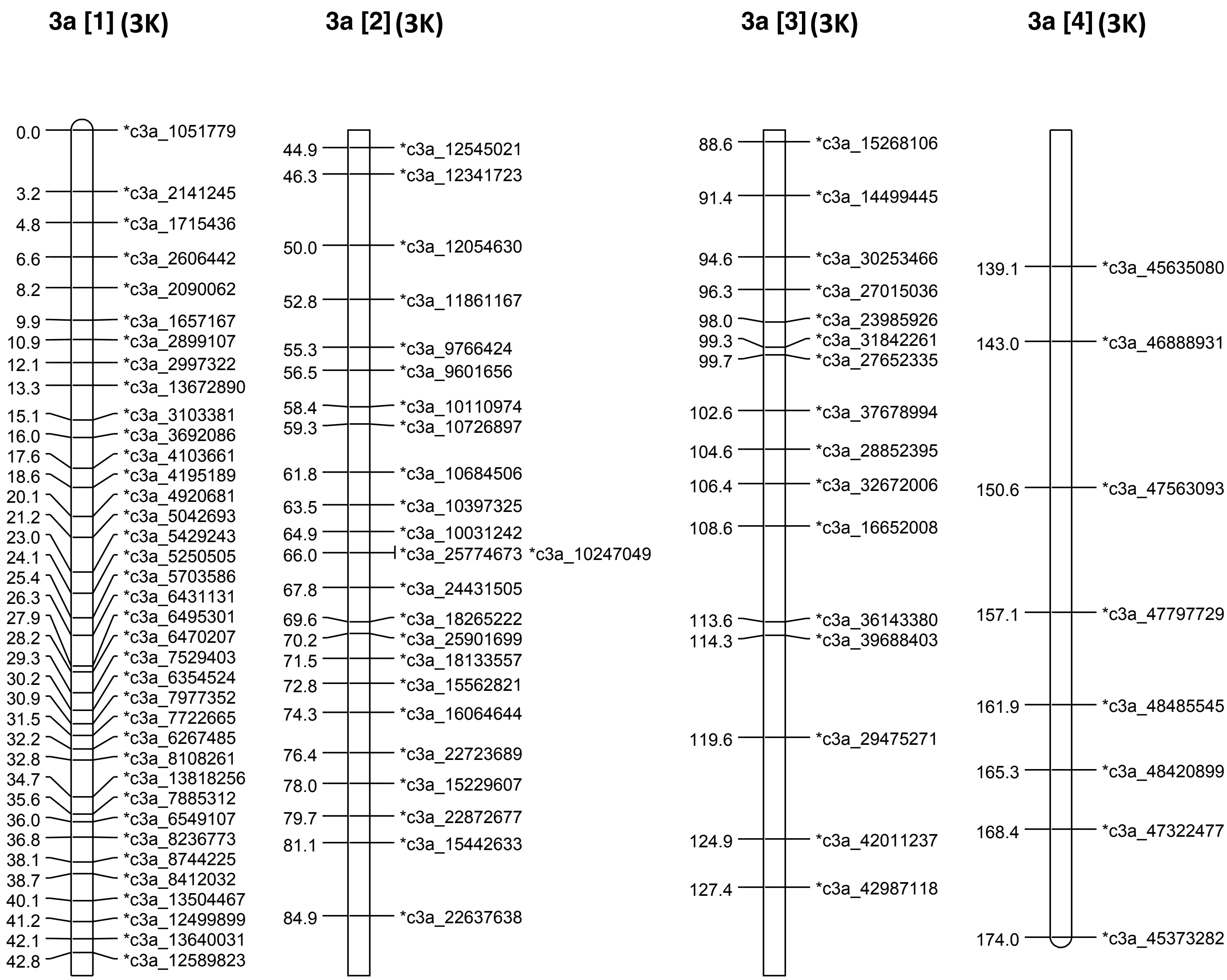
3a [1] (3K)
3a [2] (3K)
3a [3] (3K)
3a [4] (3K)
0.0 *c3a_1051779
3.2 *c3a_2141245
4.8 *c3a_1715436
6.6 *c3a_2606442
8.2 *c3a_2090062
9.9 *c3a_1657167
10.9 *c3a_2899107
12.1 *c3a_2997322
13.3 *c3a_13672890
15.1 *c3a_3103381
16.0 *c3a_3692086
17.6 *c3a_4103661
18.6 *c3a_4195189
20.1 *c3a_4920681
21.2 *c3a_5042693
23.0 *c3a_5429243
24.1 *c3a_5250505
25.4 *c3a_5703586
26.3 *c3a_6431131
27.9 *c3a_6495301
28.2 *c3a_6470207
29.3 *c3a_7529403
30.2 *c3a_6354524
30.9 *c3a_7977352
31.5 *c3a_7722665
32.2 *c3a_6267485
32.8 *c3a_8108261
34.7 *c3a_13818256
35.6 *c3a_7885312
36.0 *c3a_6549107
36.8 *c3a_8236773
38.1 *c3a_8744225
38.7 *c3a_8412032
40.1 *c3a_13504467
41.2 *c3a_12499899
42.1 *c3a_13640031
42.8 *c3a_12589823
44.9 *c3a_12545021
46.3 *c3a_12341723
50.0 *c3a_12054630
52.8 *c3a_11861167
55.3 *c3a_9766424
56.5 *c3a_9601656
58.4 *c3a_10110974
59.3 *c3a_10726897
61.8 *c3a_10684506
63.5 *c3a_10397325
64.9 *c3a_10031242
66.0 *c3a_25774673 *c3a_10247049
67.8 *c3a_24431505
69.6 *c3a_18265222
70.2 *c3a_25901699
71.5 *c3a_18133557
72.8 *c3a_15562821
74.3 *c3a_16064644
76.4 *c3a_22723689
78.0 *c3a_15229607
79.7 *c3a_22872677
81.1 *c3a_15442633
84.9 *c3a_22637638
88.6 *c3a_15268106
91.4 *c3a_14499445
94.6 *c3a_30253466
96.3 *c3a_27015036
98.0 *c3a_23985926
99.3 *c3a_31842261
99.7 *c3a_27652335
102.6 *c3a_37678994
104.6 *c3a_28852395
106.4 *c3a_32672006
108.6 *c3a_16652008
113.6 *c3a_36143380
114.3 *c3a_39688403
119.6 *c3a_29475271
124.9 *c3a_42011237
127.4 *c3a_42987118
139.1 *c3a_45635080
143.0 *c3a_46888931
150.6 *c3a_47563093
157.1 *c3a_47797729
161.9 *c3a_48485545
165.3 *c3a_48420899
168.4 *c3a_47322477
174.0 *c3a_45373282

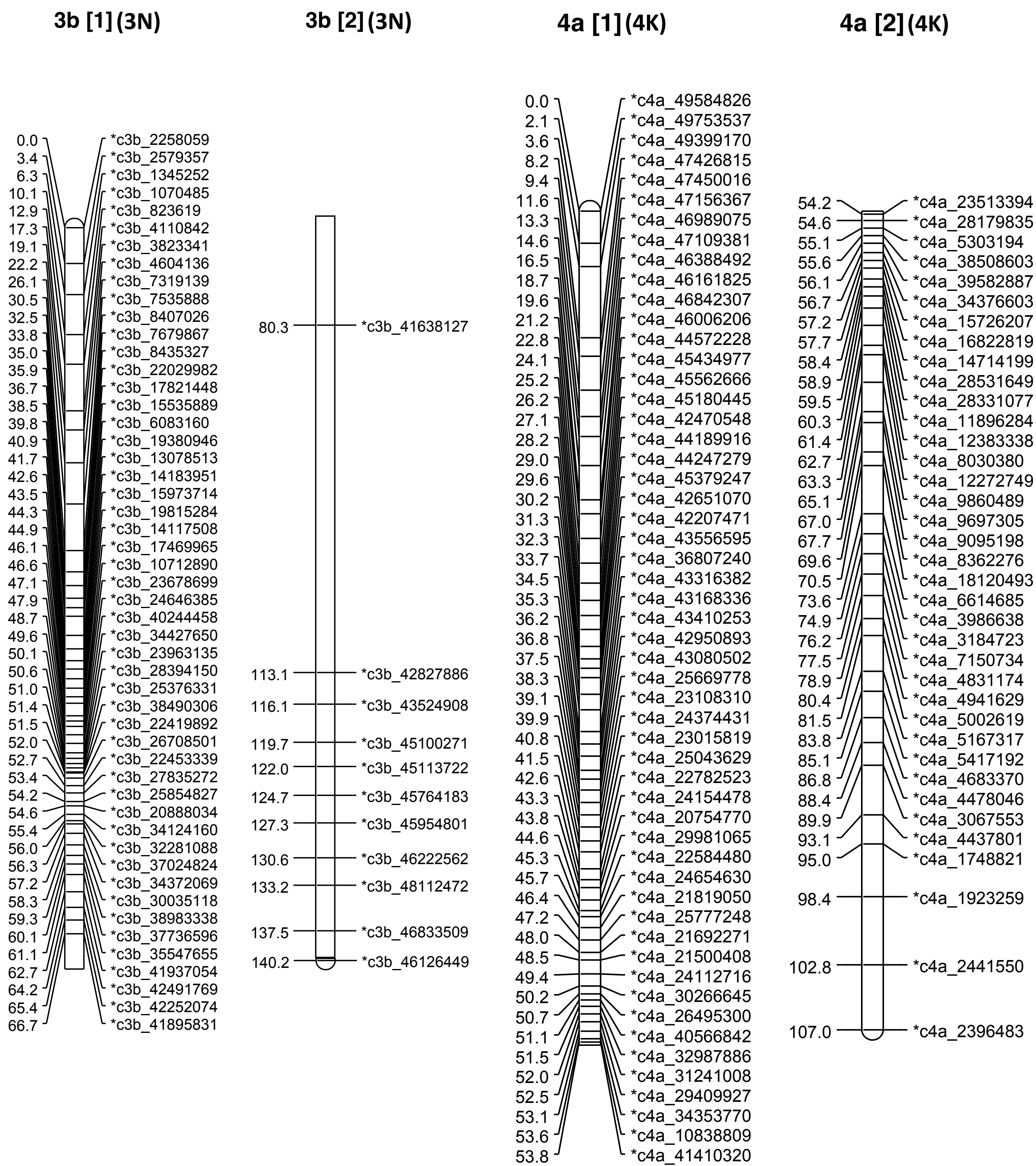
3b [1] (3N)
0.0 *c3b_2258059
3.4 *c3b_2579357
6.3 *c3b_1345252
10.1 *c3b_1070485
12.9 *c3b_823619
17.3 *c3b_4110842
19.1 *c3b_3823341
22.2 *c3b_4604136
26.1 *c3b_7319139
30.5 *c3b_7535888
32.5 *c3b_8407026
33.8 *c3b_7679867
35.0 *c3b_8435327
35.9 *c3b_22029982
36.7 *c3b_17821448
38.5 *c3b_15535889
39.8 *c3b_6083160
40.9 *c3b_19380946
41.7 *c3b_13078513
42.6 *c3b_14183951
43.5 *c3b_15973714
44.3 *c3b_19815284
44.9 *c3b_14117508
46.1 *c3b_17469965
46.6 *c3b_10712890
47.1 *c3b_23678699
47.9 *c3b_24646385
48.7 *c3b_40244458
49.6 *c3b_34427650
50.1 *c3b_23963135
50.6 *c3b_28394150
51.0 *c3b_25376331
51.4 *c3b_38490306
51.5 *c3b_22419892
52.0 *c3b_26708501
52.7 *c3b_22453339
53.4 *c3b_27835272
54.2 *c3b_25854827
54.6 *c3b_20888034
55.4 *c3b_34124160
56.0 *c3b_32281088
56.3 *c3b_37024824
57.2 *c3b_34372069
58.3 *c3b_30035118
59.3 *c3b_38983338
60.1 *c3b_37736596
61.1 *c3b_35547655
62.7 *c3b_41937054
64.2 *c3b_42491769
65.4 *c3b_42252074
66.7 *c3b_41895831
3b [2] (3N)
80.3 *c3b_41638127
113.1 *c3b_42827886
116.1 *c3b_43524908
119.7 *c3b_45100271
122.0 *c3b_45113722
124.7 *c3b_45764183
127.3 *c3b_45954801
130.6 *c3b_46222562
133.2 *c3b_48112472
137.5 *c3b_46833509
140.2 *c3b_46126449
4a [1] (4K)
0.0 *c4a_49584826
2.1 *c4a_49753537
3.6 *c4a_49399170
8.2 *c4a_47426815
9.4 *c4a_47450016
11.6 *c4a_47156367
13.3 *c4a_46989075
14.6 *c4a_47109381
16.5 *c4a_46388492
18.7 *c4a_46161825
19.6 *c4a_46842307
21.2 *c4a_46006206
22.8 *c4a_44572228
24.1 *c4a_45434977
25.2 *c4a_45562666
26.2 *c4a_45180445
27.1 *c4a_42470548
28.2 *c4a_44189916
29.0 *c4a_44247279
29.6 *c4a_45379247
30.2 *c4a_42651070
31.3 *c4a_42207471
32.3 *c4a_43556595
33.7 *c4a_36807240
34.5 *c4a_43316382
35.3 *c4a_43168336
36.2 *c4a_43410253
36.8 *c4a_42950893
37.5 *c4a_43080502
38.3 *c4a_25669778
39.1 *c4a_23108310
39.9 *c4a_24374431
40.8 *c4a_23015819
41.5 *c4a_25043629
42.6 *c4a_22782523
43.3 *c4a_24154478
43.8 *c4a_20754770
44.6 *c4a_29981065
45.3 *c4a_22584480
45.7 *c4a_24654630
46.4 *c4a_21819050
47.2 *c4a_25777248
48.0 *c4a_21692271
48.5 *c4a_21500408
49.4 *c4a_24112716
50.2 *c4a_30266645
50.7 *c4a_26495300
51.1 *c4a_40566842
51.5 *c4a_32987886
52.0 *c4a_31241008
52.5 *c4a_29409927
53.1 *c4a_34353770
53.6 *c4a_10838809
53.8 *c4a_41410320
4a [2] (4K)
54.2 *c4a_23513394
54.6 *c4a_28179835
55.1 *c4a_5303194
55.6 *c4a_38508603
56.1 *c4a_39582887
56.7 *c4a_34376603
57.2 *c4a_15726207
57.7 *c4a_16822819
58.4 *c4a_14714199
58.9 *c4a_28531649
59.5 *c4a_28331077
60.3 *c4a_11896284
61.4 *c4a_12383338
62.7 *c4a_8030380
63.3 *c4a_12272749
65.1 *c4a_9860489
67.0 *c4a_9697305
67.7 *c4a_9095198
69.6 *c4a_8362276
70.5 *c4a_18120493
73.6 *c4a_6614685
74.9 *c4a_3986638
76.2 *c4a_3184723
77.5 *c4a_7150734
78.9 *c4a_4831174
80.4 *c4a_4941629
81.5 *c4a_5002619
83.8 *c4a_5167317
85.1 *c4a_5417192
86.8 *c4a_4683370
88.4 *c4a_4478046
89.9 *c4a_3067553
93.1 *c4a_4437801
95.0 *c4a_1748821
98.4 *c4a_1923259
102.8 *c4a_2441550
107.0 *c4a_2396483

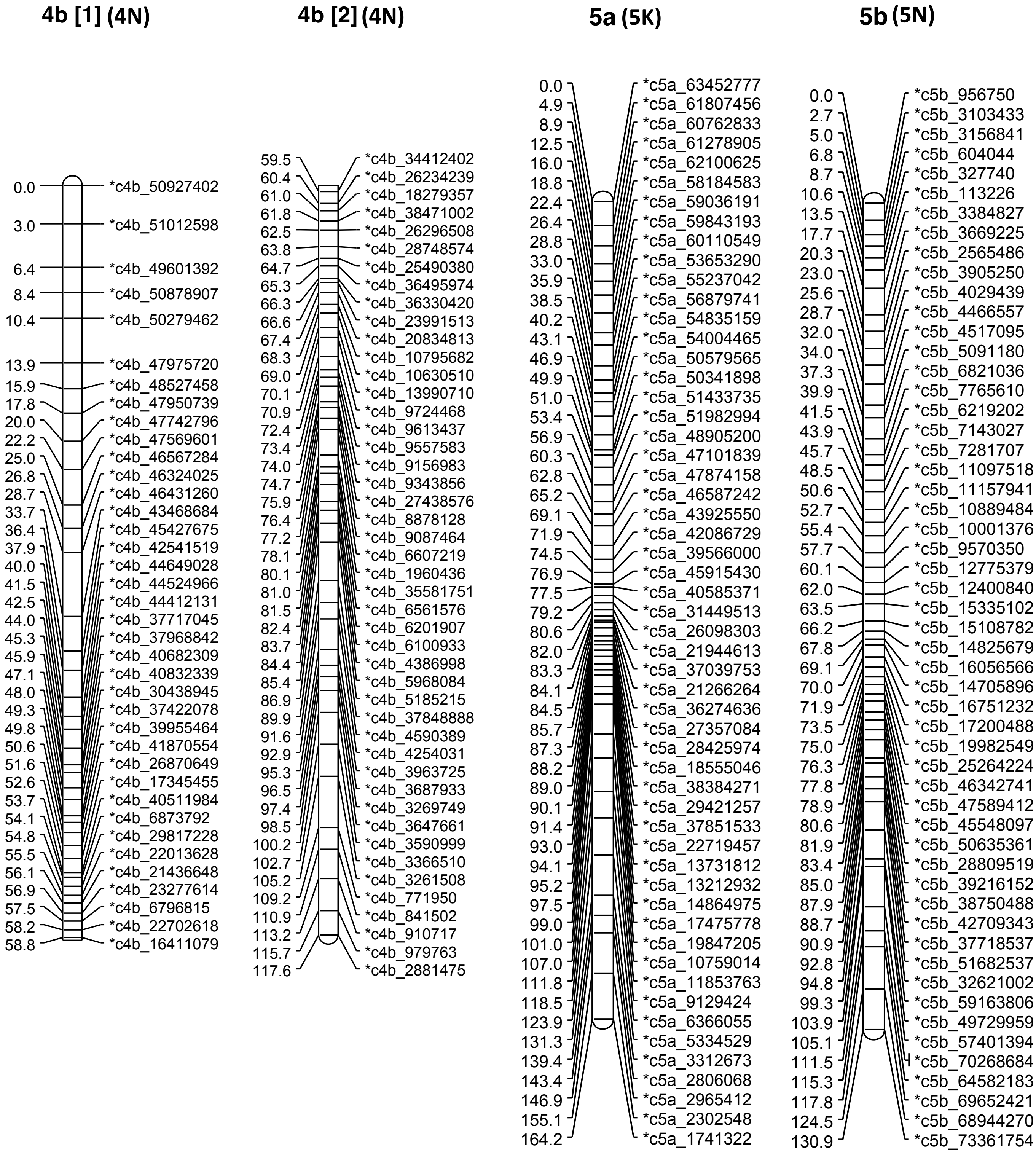
4b [1] (4N)
0.0 *c4b_50927402
3.0 *c4b_51012598
6.4 *c4b_49601392
8.4 *c4b_50878907
10.4 *c4b_50279462
13.9 *c4b_47975720
15.9 *c4b_48527458
17.8 *c4b_47950739
20.0 *c4b_47742796
22.2 *c4b_47569601
25.0 *c4b_46567284
26.8 *c4b_46324025
28.7 *c4b_46431260
33.7 *c4b_43468684
36.4 *c4b_45427675
37.9 *c4b_42541519
40.0 *c4b_44649028
41.5 *c4b_44524966
42.5 *c4b_44412131
44.0 *c4b_37717045
45.3 *c4b_37968842
45.9 *c4b_40682309
47.1 *c4b_40832339
48.0 *c4b_30438945
49.3 *c4b_37422078
49.8 *c4b_39955464
50.6 *c4b_41870554
51.6 *c4b_26870649
52.6 *c4b_17345455
53.7 *c4b_40511984
54.1 *c4b_6873792
54.8 *c4b_29817228
55.5 *c4b_22013628
56.1 *c4b_21436648
56.9 *c4b_23277614
57.5 *c4b_6796815
58.2 *c4b_22702618
58.8 *c4b_16411079
4b [2] (4N)
59.5 *c4b_34412402
60.4 *c4b_26234239
61.0 *c4b_18279357
61.8 *c4b_38471002
62.5 *c4b_26296508
63.8 *c4b_28748574
64.7 *c4b_25490380
65.3 *c4b_36495974
66.3 *c4b_36330420
66.6 *c4b_23991513
67.4 *c4b_20834813
68.3 *c4b_10795682
69.0 *c4b_10630510
70.1 *c4b_13990710
70.9 *c4b_9724468
72.4 *c4b_9613437
73.4 *c4b_9557583
74.0 *c4b_9156983
74.7 *c4b_9343856
75.9 *c4b_27438576
76.4 *c4b_8878128
77.2 *c4b_9087464
78.1 *c4b_6607219
80.1 *c4b_1960436
81.0 *c4b_35581751
81.5 *c4b_6561576
82.4 *c4b_6201907
83.7 *c4b_6100933
84.4 *c4b_4386998
85.4 *c4b_5968084
86.9 *c4b_5185215
89.9 *c4b_37848888
91.6 *c4b_4590389
92.9 *c4b_4254031
95.3 *c4b_3963725
96.5 *c4b_3687933
97.4 *c4b_3269749
98.5 *c4b_3647661
100.2 *c4b_3590999
102.7 *c4b_3366510
105.2 *c4b_3261508
109.2 *c4b_771950
110.9 *c4b_841502
113.2 *c4b_910717
115.7 *c4b_979763
117.6 *c4b_2881475
5a (5K)
0.0 *c5a_63452777
4.9 *c5a_61807456
8.9 *c5a_60762833
12.5 *c5a_61278905
16.0 *c5a_62100625
18.8 *c5a_58184583
22.4 *c5a_59036191
26.4 *c5a_59843193
28.8 *c5a_60110549
33.0 *c5a_53653290
35.9 *c5a_55237042
38.5 *c5a_56879741
40.2 *c5a_54835159
43.1 *c5a_54004465
46.9 *c5a_50579565
49.9 *c5a_50341898
51.0 *c5a_51433735
53.4 *c5a_51982994
56.9 *c5a_48905200
60.3 *c5a_47101839
62.8 *c5a_47874158
65.2 *c5a_46587242
69.1 *c5a_43925550
71.9 *c5a_42086729
74.5 *c5a_39566000
76.9 *c5a_45915430
77.5 *c5a_40585371
79.2 *c5a_31449513
80.6 *c5a_26098303
82.0 *c5a_21944613
83.3 *c5a_37039753
84.1 *c5a_21266264
84.5 *c5a_36274636
85.7 *c5a_27357084
87.3 *c5a_28425974
88.2 *c5a_18555046
89.0 *c5a_38384271
90.1 *c5a_29421257
91.4 *c5a_37851533
93.0 *c5a_22719457
94.1 *c5a_13731812
95.2 *c5a_13212932
97.5 *c5a_14864975
99.0 *c5a_17475778
101.0 *c5a_19847205
107.0 *c5a_10759014
111.8 *c5a_11853763
118.5 *c5a_9129424
123.9 *c5a_6366055
131.3 *c5a_5334529
139.4 *c5a_3312673
143.4 *c5a_2806068
146.9 *c5a_2965412
155.1 *c5a_2302548
164.2 *c5a_1741322
5b (5N)
0.0 *c5b_956750
2.7 *c5b_3103433
5.0 *c5b_3156841
6.8 *c5b_604044
8.7 *c5b_327740
10.6 *c5b_113226
13.5 *c5b_3384827
17.7 *c5b_3669225
20.3 *c5b_2565486
23.0 *c5b_3905250
25.6 *c5b_4029439
28.7 *c5b_4466557
32.0 *c5b_4517095
34.0 *c5b_5091180
37.3 *c5b_6821036
39.9 *c5b_7765610
41.5 *c5b_6219202
43.9 *c5b_7143027
45.7 *c5b_7281707
48.5 *c5b_11097518
50.6 *c5b_11157941
52.7 *c5b_10889484
55.4 *c5b_10001376
57.7 *c5b_9570350
60.1 *c5b_12775379
62.0 *c5b_12400840
63.5 *c5b_15335102
66.2 *c5b_15108782
67.8 *c5b_14825679
69.1 *c5b_16056566
70.0 *c5b_14705896
71.9 *c5b_16751232
73.5 *c5b_17200488
75.0 *c5b_19982549
76.3 *c5b_25264224
77.8 *c5b_46342741
78.9 *c5b_47589412
80.6 *c5b_45548097
81.9 *c5b_50635361
83.4 *c5b_28809519
85.0 *c5b_39216152
87.9 *c5b_38750488
88.7 *c5b_42709343
90.9 *c5b_37718537
92.8 *c5b_51682537
94.8 *c5b_32621002
99.3 *c5b_59163806
103.9 *c5b_49729959
105.1 *c5b_57401394
111.5 *c5b_70268684
115.3 *c5b_64582183
117.8 *c5b_69652421
124.5 *c5b_68944270
130.9 *c5b_73361754

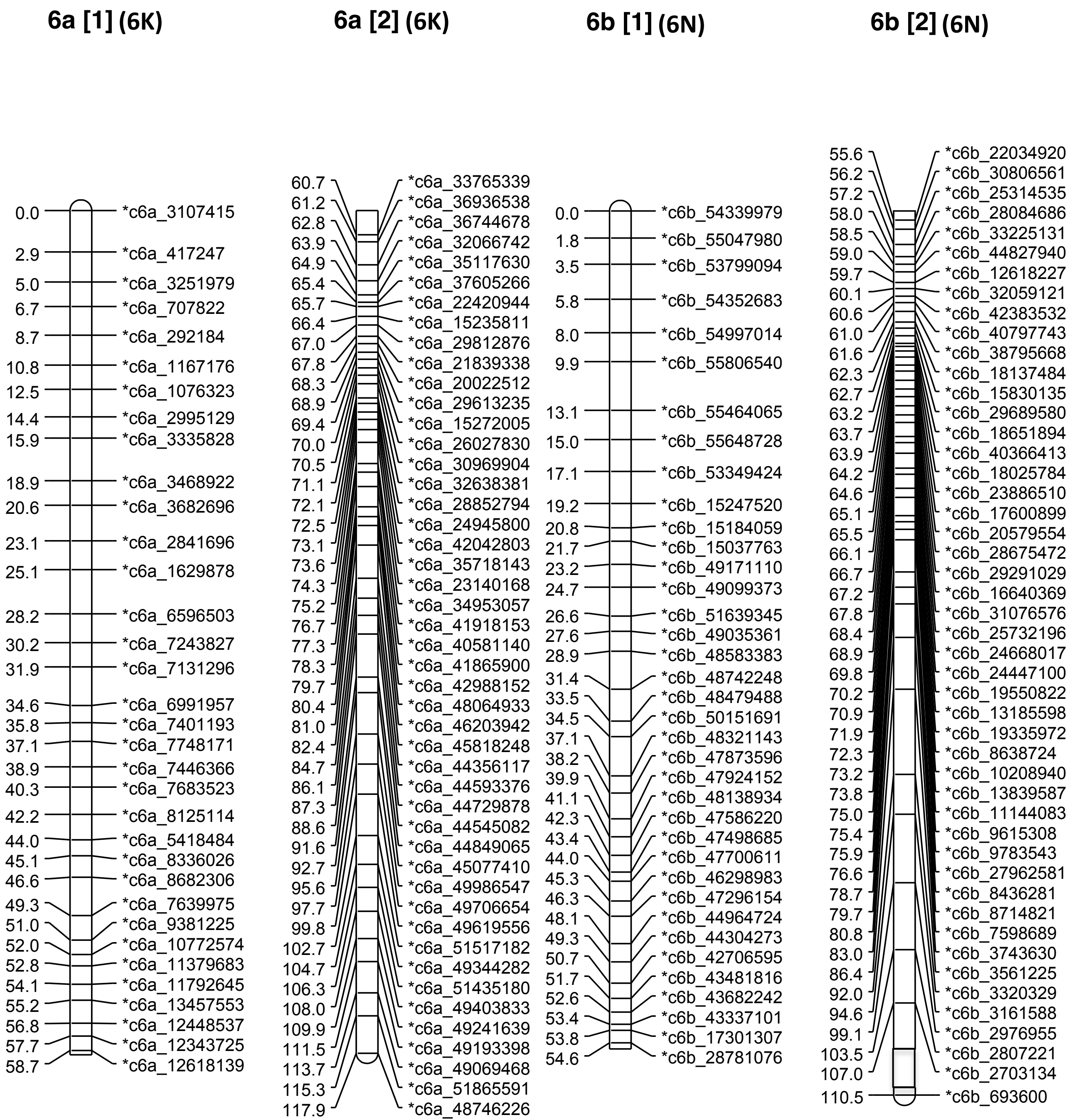

6a [1] (6K)
0.0 *c6a_3107415
2.9 *c6a_417247
5.0 *c6a_3251979
6.7 *c6a_707822
8.7 *c6a_292184
10.8 *c6a_1167176
12.5 *c6a_1076323
14.4 *c6a_2995129
15.9 *c6a_3335828
18.9 *c6a_3468922
20.6 *c6a_3682696
23.1 *c6a_2841696
25.1 *c6a_1629878
28.2 *c6a_6596503
30.2 *c6a_7243827
31.9 *c6a_7131296
34.6 *c6a_6991957
35.8 *c6a_7401193
37.1 *c6a_7748171
38.9 *c6a_7446366
40.3 *c6a_7683523
42.2 *c6a_8125114
44.0 *c6a_5418484
45.1 *c6a_8336026
46.6 *c6a_8682306
49.3 *c6a_7639975
51.0 *c6a_9381225
52.0 *c6a_10772574
52.8 *c6a_11379683
54.1 *c6a_11792645
55.2 *c6a_13457553
56.8 *c6a_12448537
57.7 *c6a_12343725
58.7 *c6a_12618139
6a [2] (6K)
60.7 *c6a_33765339
61.2 *c6a_36936538
62.8 *c6a_36744678
63.9 *c6a_32066742
64.9 *c6a_35117630
65.4 *c6a_37605266
65.7 *c6a_22420944
66.4 *c6a_15235811
67.0 *c6a_29812876
67.8 *c6a_21839338
68.3 *c6a_20022512
68.9 *c6a_29613235
69.4 *c6a_15272005
70.0 *c6a_26027830
70.5 *c6a_30969904
71.1 *c6a_32638381
72.1 *c6a_28852794
72.5 *c6a_24945800
73.1 *c6a_42042803
73.6 *c6a_35718143
74.3 *c6a_23140168
75.2 *c6a_34953057
76.7 *c6a_41918153
77.3 *c6a_40581140
78.3 *c6a_41865900
79.7 *c6a_42988152
80.4 *c6a_48064933
81.0 *c6a_46203942
82.4 *c6a_45818248
84.7 *c6a_44356117
86.1 *c6a_44593376
87.3 *c6a_44729878
88.6 *c6a_44545082
91.6 *c6a_44849065
92.7 *c6a_45077410
95.6 *c6a_49986547
97.7 *c6a_49706654
99.8 *c6a_49619556
102.7 *c6a_51517182
104.7 *c6a_49344282
106.3 *c6a_51435180
108.0 *c6a_49403833
109.9 *c6a_49241639
111.5 *c6a_49193398
113.7 *c6a_49069468
115.3 *c6a_51865591
117.9 *c6a_48746226
6b [1] (6N)
0.0 *c6b_54339979
1.8 *c6b_55047980
3.5 *c6b_53799094
5.8 *c6b_54352683
8.0 *c6b_54997014
9.9 *c6b_55806540
13.1 *c6b_55464065
15.0 *c6b_55648728
17.1 *c6b_53349424
19.2 *c6b_15247520
20.8 *c6b_15184059
21.7 *c6b_15037763
23.2 *c6b_49171110
24.7 *c6b_49099373
26.6 *c6b_51639345
27.6 *c6b_49035361
28.9 *c6b_48583383
31.4 *c6b_48742248
33.5 *c6b_48479488
34.5 *c6b_50151691
37.1 *c6b_48321143
38.2 *c6b_47873596
39.9 *c6b_47924152
41.1 *c6b_48138934
42.3 *c6b_47586220
43.4 *c6b_47498685
44.0 *c6b_47700611
45.3 *c6b_46298983
46.3 *c6b_47296154
48.1 *c6b_44964724
49.3 *c6b_44304273
50.7 *c6b_42706595
51.7 *c6b_43481816
52.6 *c6b_43682242
53.4 *c6b_43337101
53.8 *c6b_17301307
54.6 *c6b_28781076
6b [2] (6N)
55.6 *c6b_22034920
56.2 *c6b_30806561
57.2 *c6b_25314535
58.0 *c6b_28084686
58.5 *c6b_33225131
59.0 *c6b_44827940
59.7 *c6b_12618227
60.1 *c6b_32059121
60.6 *c6b_42383532
61.0 *c6b_40797743
61.6 *c6b_38795668
62.3 *c6b_18137484
62.7 *c6b_15830135
63.2 *c6b_29689580
63.7 *c6b_18651894
63.9 *c6b_40366413
64.2 *c6b_18025784
64.6 *c6b_23886510
65.1 *c6b_17600899
65.5 *c6b_20579554
66.1 *c6b_28675472
66.7 *c6b_29291029
67.2 *c6b_16640369
67.8 *c6b_31076576
68.4 *c6b_25732196
68.9 *c6b_24668017
69.8 *c6b_24447100
70.2 *c6b_19550822
70.9 *c6b_13185598
71.9 *c6b_19335972
72.3 *c6b_8638724
73.2 *c6b_10208940
73.8 *c6b_13839587
75.0 *c6b_11144083
75.4 *c6b_9615308
75.9 *c6b_9783543
76.6 *c6b_27962581
78.7 *c6b_8436281
79.7 *c6b_8714821
80.8 *c6b_7598689
83.0 *c6b_3743630
86.4 *c6b_3561225
92.0 *c6b_3320329
94.6 *c6b_3161588
99.1 *c6b_2976955
103.5 *c6b_2807221
107.0 *c6b_2703134
110.5 *c6b_693600

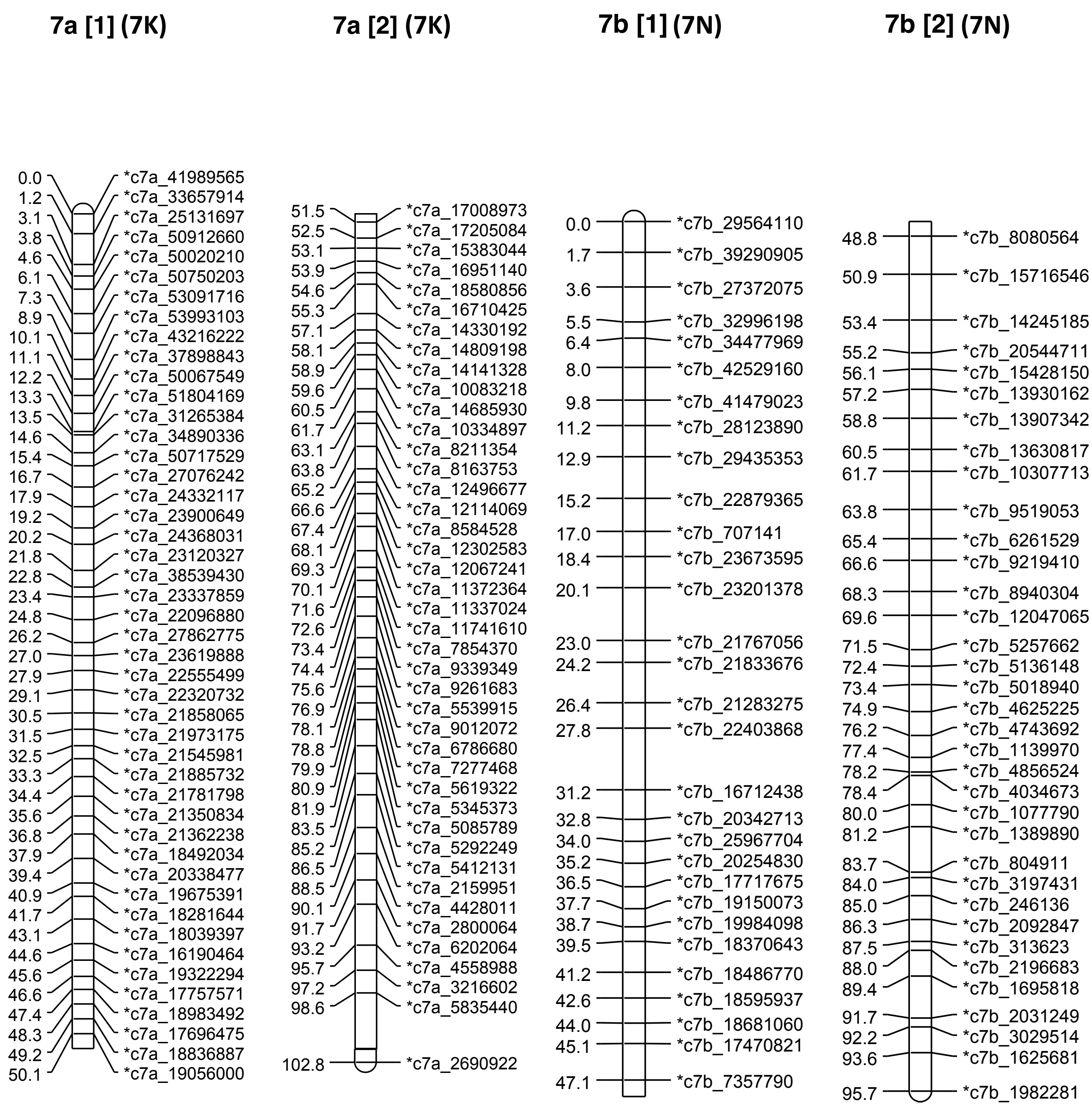

7a [1] (7K)
0.0 *c7a_41989565
1.2 *c7a_33657914
3.1 *c7a_25131697
3.8 *c7a_50912660
4.6 *c7a_50020210
6.1 *c7a_50750203
7.3 *c7a_53091716
8.9 *c7a_53993103
10.1 *c7a_43216222
11.1 *c7a_37898843
12.2 *c7a_50067549
13.3 *c7a_51804169
13.5 *c7a_31265384
14.6 *c7a_34890336
15.4 *c7a_50717529
16.7 *c7a_27076242
17.9 *c7a_24332117
19.2 *c7a_23900649
20.2 *c7a_24368031
21.8 *c7a_23120327
22.8 *c7a_38539430
23.4 *c7a_23337859
24.8 *c7a_22096880
26.2 *c7a_27862775
27.0 *c7a_23619888
27.9 *c7a_22555499
29.1 *c7a_22320732
30.5 *c7a_21858065
31.5 *c7a_21973175
32.5 *c7a_21545981
33.3 *c7a_21885732
34.4 *c7a_21781798
35.6 *c7a_21350834
36.8 *c7a_21362238
37.9 *c7a_18492034
39.4 *c7a_20338477
40.9 *c7a_19675391
41.7 *c7a_18281644
43.1 *c7a_18039397
44.6 *c7a_16190464
45.6 *c7a_19322294
46.6 *c7a_17757571
47.4 *c7a_18983492
48.3 *c7a_17696475
49.2 *c7a_18836887
50.1 *c7a_19056000
7a [2] (7K)
51.5 *c7a_17008973
52.5 *c7a_17205084
53.1 *c7a_15383044
53.9 *c7a_16951140
54.6 *c7a_18580856
55.3 *c7a_16710425
57.1 *c7a_14330192
58.1 *c7a_14809198
58.9 *c7a_14141328
59.6 *c7a_10083218
60.5 *c7a_14685930
61.7 *c7a_10334897
63.1 *c7a_8211354
63.8 *c7a_8163753
65.2 *c7a_12496677
66.6 *c7a_12114069
67.4 *c7a_8584528
68.1 *c7a_12302583
69.3 *c7a_12067241
70.1 *c7a_11372364
71.6 *c7a_11337024
72.6 *c7a_11741610
73.4 *c7a_7854370
74.4 *c7a_9339349
75.6 *c7a_9261683
76.9 *c7a_5539915
78.1 *c7a_9012072
78.8 *c7a_6786680
79.9 *c7a_7277468
80.9 *c7a_5619322
81.9 *c7a_5345373
83.5 *c7a_5085789
85.2 *c7a_5292249
86.5 *c7a_5412131
88.5 *c7a_2159951
90.1 *c7a_4428011
91.7 *c7a_2800064
93.2 *c7a_6202064
95.7 *c7a_4558988
97.2 *c7a_3216602
98.6 *c7a_5835440
102.8 *c7a_2690922
7b [1] (7N)
0.0 *c7b_29564110
1.7 *c7b_39290905
3.6 *c7b_27372075
5.5 *c7b_32996198
6.4 *c7b_34477969
8.0 *c7b_42529160
9.8 *c7b_41479023
11.2 *c7b_28123890
12.9 *c7b_29435353
15.2 *c7b_22879365
17.0 *c7b_707141
18.4 *c7b_23673595
20.1 *c7b_23201378
23.0 *c7b_21767056
24.2 *c7b_21833676
26.4 *c7b_21283275
27.8 *c7b_22403868
31.2 *c7b_16712438
32.8 *c7b_20342713
34.0 *c7b_25967704
35.2 *c7b_20254830
36.5 *c7b_17717675
37.7 *c7b_19150073
38.7 *c7b_19984098
39.5 *c7b_18370643
41.2 *c7b_18486770
42.6 *c7b_18595937
44.0 *c7b_18681060
45.1 *c7b_17470821
47.1 *c7b_7357790
7b [2] (7N)
48.8 *c7b_8080564
50.9 *c7b_15716546
53.4 *c7b_14245185
55.2 *c7b_20544711
56.1 *c7b_15428150
57.2 *c7b_13930162
58.8 *c7b_13907342
60.5 *c7b_13630817
61.7 *c7b_10307713
63.8 *c7b_9519053
65.4 *c7b_6261529
66.6 *c7b_9219410
68.3 *c7b_8940304
69.6 *c7b_12047065
71.5 *c7b_5257662
72.4 *c7b_5136148
73.4 *c7b_5018940
74.9 *c7b_4625225
76.2 *c7b_4743692
77.4 *c7b_1139970
78.2 *c7b_4856524
78.4 *c7b_4034673
80.0 *c7b_1077790
81.2 *c7b_1389890
83.7 *c7b_804911
84.0 *c7b_3197431
85.0 *c7b_246136
86.3 *c7b_2092847
87.5 *c7b_313623
88.0 *c7b_2196683
89.4 *c7b_1695818
91.7 *c7b_2031249
92.2 *c7b_3029514
93.6 *c7b_1625681
95.7 *c7b_1982281

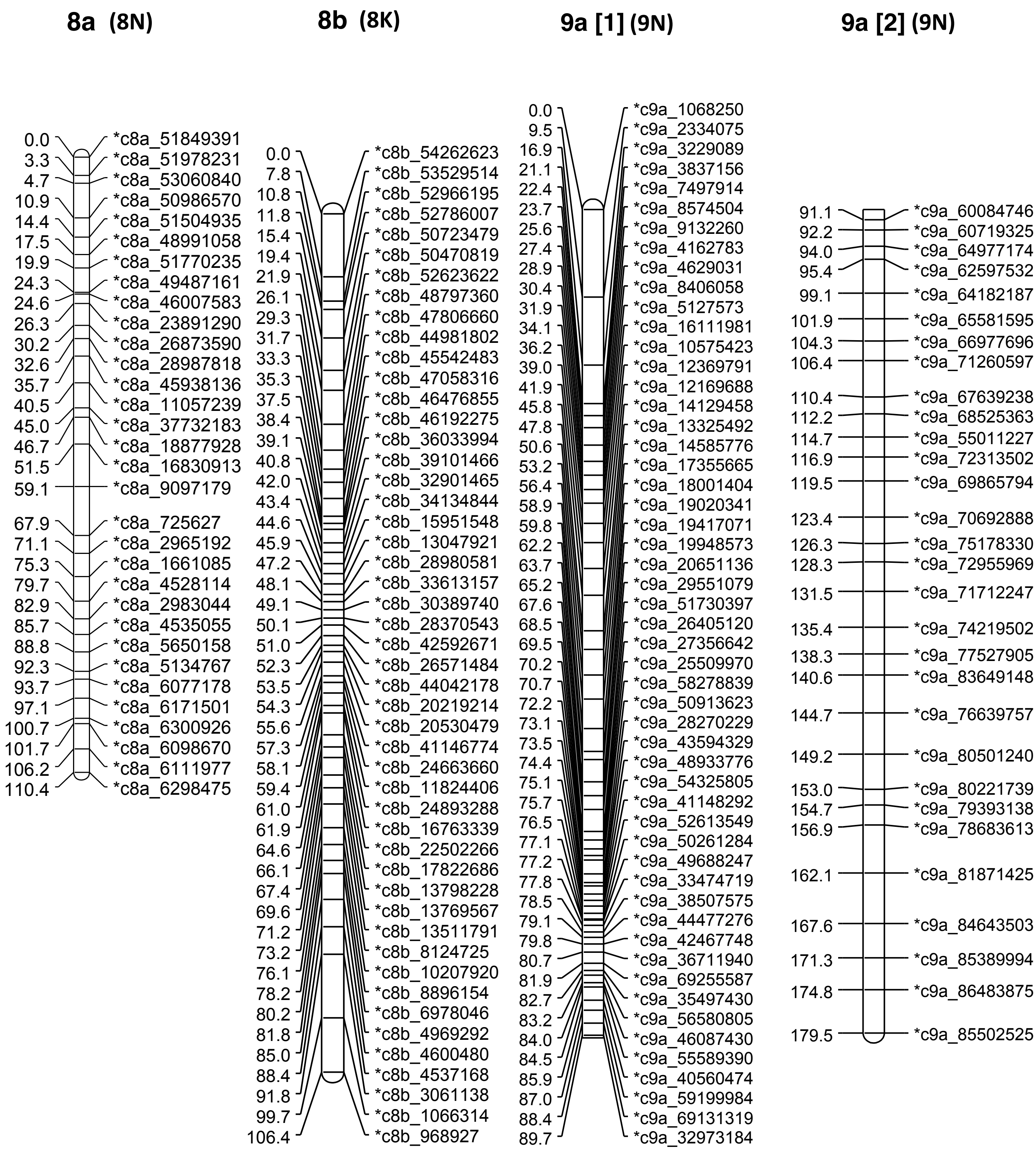
8a (8N)
0.0 *c8a_51849391
3.3 *c8a_51978231
4.7 *c8a_53060840
10.9 *c8a_50986570
14.4 *c8a_51504935
17.5 *c8a_48991058
19.9 *c8a_51770235
24.3 *c8a_49487161
24.6 *c8a_46007583
26.3 *c8a_23891290
30.2 *c8a_26873590
32.6 *c8a_28987818
35.7 *c8a_45938136
40.5 *c8a_11057239
45.0 *c8a_37732183
46.7 *c8a_18877928
51.5 *c8a_16830913
59.1 *c8a_9097179
67.9 *c8a_725627
71.1 *c8a_2965192
75.3 *c8a_1661085
79.7 *c8a_4528114
82.9 *c8a_2983044
85.7 *c8a_4535055
88.8 *c8a_5650158
92.3 *c8a_5134767
93.7 *c8a_6077178
97.1 *c8a_6171501
100.7 *c8a_6300926
101.7 *c8a_6098670
106.2 *c8a_6111977
110.4 *c8a_6298475
8b (8K)
0.0 *c8b_54262623
7.8 *c8b_53529514
10.8 *c8b_52966195
11.8 *c8b_52786007
15.4 *c8b_50723479
19.4 *c8b_50470819
21.9 *c8b_52623622
26.1 *c8b_48797360
29.3 *c8b_47806660
31.7 *c8b_44981802
33.3 *c8b_45542483
35.3 *c8b_47058316
37.5 *c8b_46476855
38.4 *c8b_46192275
39.1 *c8b_36033994
40.8 *c8b_39101466
42.0 *c8b_32901465
43.4 *c8b_34134844
44.6 *c8b_15951548
45.9 *c8b_13047921
47.2 *c8b_28980581
48.1 *c8b_33613157
49.1 *c8b_30389740
50.1 *c8b_28370543
51.0 *c8b_42592671
52.3 *c8b_26571484
53.5 *c8b_44042178
54.3 *c8b_20219214
55.6 *c8b_20530479
57.3 *c8b_41146774
58.1 *c8b_24663660
59.4 *c8b_11824406
61.0 *c8b_24893288
61.9 *c8b_16763339
64.6 *c8b_22502266
66.1 *c8b_17822686
67.4 *c8b_13798228
69.6 *c8b_13769567
71.2 *c8b_13511791
73.2 *c8b_8124725
76.1 *c8b_10207920
78.2 *c8b_8896154
80.2 *c8b_6978046
81.8 *c8b_4969292
85.0 *c8b_4600480
88.4 *c8b_4537168
91.8 *c8b_3061138
99.7 *c8b_1066314
106.4 *c8b_968927
9a [1] (9N)
0.0 *c9a_1068250
9.5 *c9a_2334075
16.9 *c9a_3229089
21.1 *c9a_3837156
22.4 *c9a_7497914
23.7 *c9a_8574504
25.6 *c9a_9132260
27.4 *c9a_4162783
28.9 *c9a_4629031
30.4 *c9a_8406058
31.9 *c9a_5127573
34.1 *c9a_16111981
36.2 *c9a_10575423
39.0 *c9a_12369791
41.9 *c9a_12169688
45.8 *c9a_14129458
47.8 *c9a_13325492
50.6 *c9a_14585776
53.2 *c9a_17355665
56.4 *c9a_18001404
58.9 *c9a_19020341
59.8 *c9a_19417071
62.2 *c9a_19948573
63.7 *c9a_20651136
65.2 *c9a_29551079
67.6 *c9a_51730397
68.5 *c9a_26405120
69.5 *c9a_27356642
70.2 *c9a_25509970
70.7 *c9a_58278839
72.2 *c9a_50913623
73.1 *c9a_28270229
73.5 *c9a_43594329
74.4 *c9a_48933776
75.1 *c9a_54325805
75.7 *c9a_41148292
76.5 *c9a_52613549
77.1 *c9a_50261284
77.2 *c9a_49688247
77.8 *c9a_33474719
78.5 *c9a_38507575
79.1 *c9a_44477276
79.8 *c9a_42467748
80.7 *c9a_36711940
81.9 *c9a_69255587
82.7 *c9a_35497430
83.2 *c9a_56580805
84.0 *c9a_46087430
84.5 *c9a_55589390
85.9 *c9a_40560474
87.0 *c9a_59199984
88.4 *c9a_69131319
89.7 *c9a_32973184
9a [2] (9N)
91.1 *c9a_60084746
92.2 *c9a_60719325
94.0 *c9a_64977174
95.4 *c9a_62597532
99.1 *c9a_64182187
101.9 *c9a_65581595
104.3 *c9a_66977696
106.4 *c9a_71260597
110.4 *c9a_67639238
112.2 *c9a_68525363
114.7 *c9a_55011227
116.9 *c9a_72313502
119.5 *c9a_69865794
123.4 *c9a_70692888
126.3 *c9a_75178330
128.3 *c9a_72955969
131.5 *c9a_71712247
135.4 *c9a_74219502
138.3 *c9a_77527905
140.6 *c9a_83649148
144.7 *c9a_76639757
149.2 *c9a_80501240
153.0 *c9a_80221739
154.7 *c9a_79393138
156.9 *c9a_78683613
162.1 *c9a_81871425
167.6 *c9a_84643503
171.3 *c9a_85389994
174.8 *c9a_86483875
179.5 *c9a_85502525

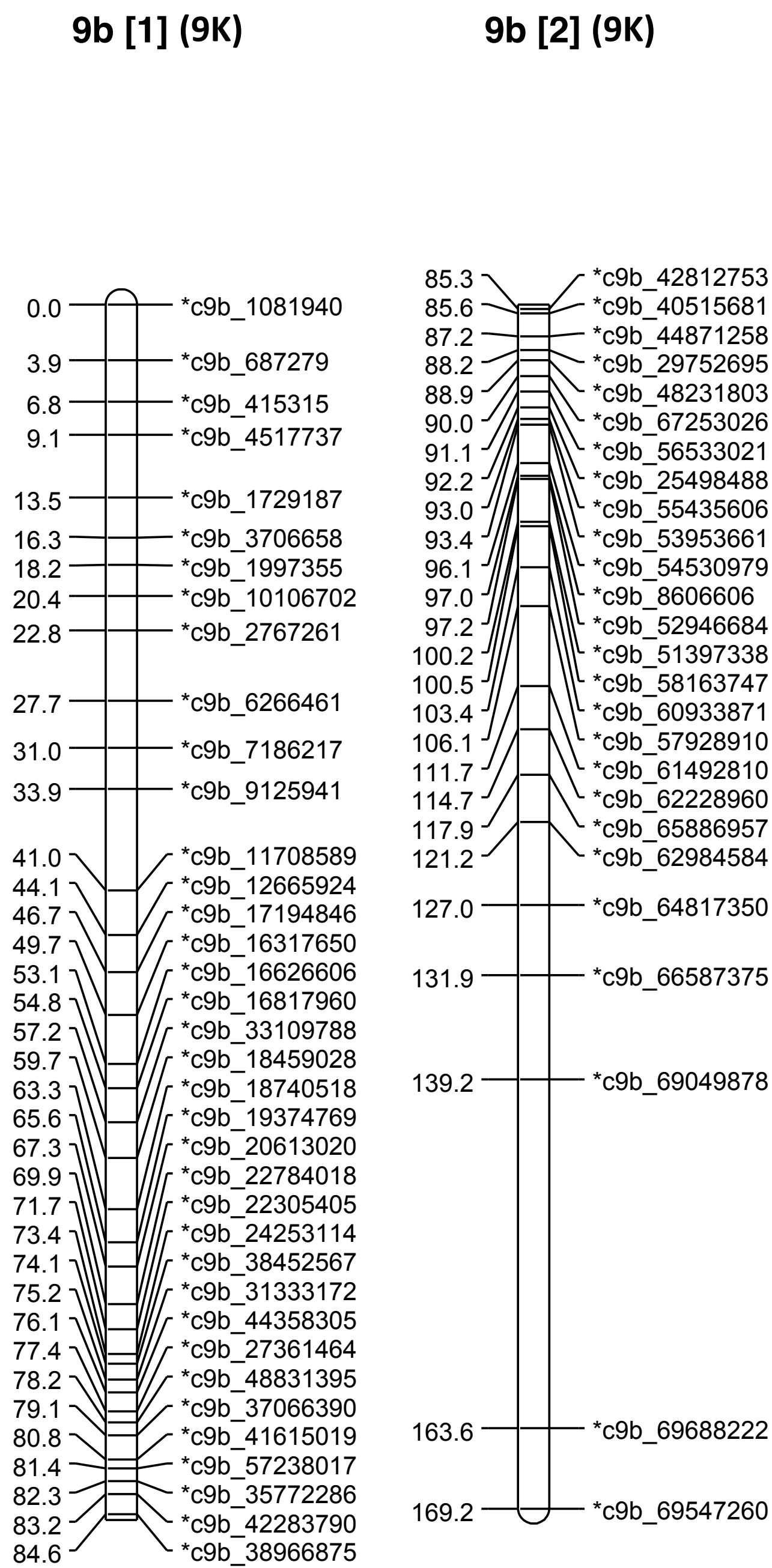
9b [1] (9K)
9b [2] (9K)
0.0 *c9b_1081940
3.9 *c9b_687279
6.8 *c9b_415315
9.1 *c9b_4517737
13.5 *c9b_1729187
16.3 *c9b_3706658
18.2 *c9b_1997355
20.4 *c9b_10106702
22.8 *c9b_2767261
27.7 *c9b_6266461
31.0 *c9b_7186217
33.9 *c9b_9125941
41.0 *c9b_11708589
44.1 *c9b_12665924
46.7 *c9b_17194846
49.7 *c9b_16317650
53.1 *c9b_16626606
54.8 *c9b_16817960
57.2 *c9b_33109788
59.7 *c9b_18459028
63.3 *c9b_18740518
65.6 *c9b_19374769
67.3 *c9b_20613020
69.9 *c9b_22784018
71.7 *c9b_22305405
73.4 *c9b_24253114
74.1 *c9b_38452567
75.2 *c9b_31333172
76.1 *c9b_44358305
77.4 *c9b_27361464
78.2 *c9b_48831395
79.1 *c9b_37066390
80.8 *c9b_41615019
81.4 *c9b_57238017
82.3 *c9b_35772286
83.2 *c9b_42283790
84.6 *c9b_38966875
85.3 *c9b_42812753
85.6 *c9b_40515681
87.2 *c9b_44871258
88.2 *c9b_29752695
88.9 *c9b_48231803
90.0 *c9b_67253026
91.1 *c9b_56533021
92.2 *c9b_25498488
93.0 *c9b_55435606
93.4 *c9b_53953661
96.1 *c9b_54530979
97.0 *c9b_8606606
97.2 *c9b_52946684
100.2 *c9b_51397338
100.5 *c9b_58163747
103.4 *c9b_60933871
106.1 *c9b_57928910
111.7 *c9b_61492810
114.7 *c9b_62228960
117.9 *c9b_65886957
121.2 *c9b_62984584
127.0 *c9b_64817350
131.9 *c9b_66587375
139.2 *c9b_69049878
163.6 *c9b_69688222
169.2 *c9b_69547260
